# Acetylation accumulates PFKFB3 in cytoplasm to promote glycolysis and protects cells from cisplatin-induced apoptosis

**DOI:** 10.1038/s41467-018-02950-5

**Published:** 2018-02-06

**Authors:** Fu-Long Li, Jin-Ping Liu, Ruo-Xuan Bao, GuoQuan Yan, Xu Feng, Yan-Ping Xu, Yi-Ping Sun, Weili Yan, Zhi-Qiang Ling, Yue Xiong, Kun-Liang Guan, Hai-Xin Yuan

**Affiliations:** 1 The Fifth People’s Hospital of Shanghai and the Molecular and Cell Biology Research Lab of the Institutes of Biomedical Sciences, Fudan University, Shanghai, 200032 China; 2 School of Life Sciences, Fudan University, Shanghai, 200032 China; 3 Institutes of Biomedical Sciences, Fudan University, Shanghai, 200032 China; 4 Lineberger Comprehensive Cancer Center, Department of Biochemistry and Biophysics, University of North Carolina at Chapel Hill, Chapel Hill, NC 27599 USA; 5 Department of Clinical Epidemiology, Children’s Hospital of Fudan University, Shanghai, 201102 China; 6 Zhejiang Cancer Research Institute, Zhejiang Province Cancer Hospital Zhejiang Cancer Center, Hangzhou, 310022 China; 7 Department of Pharmacology and Moores Cancer Center, University of California San Diego, La Jolla, CA 92093 USA

## Abstract

Enhanced glycolysis in cancer cells has been linked to cell protection from DNA damaging signals, although the mechanism is largely unknown. The 6-phosphofructo-2-kinase/fructose-2,6-biphosphatase 3 (PFKFB3) catalyzes the generation of fructose-2,6-bisphosphate, a potent allosteric stimulator of glycolysis. Intriguingly, among the four members of PFKFB family, PFKFB3 is uniquely localized in the nucleus, although the reason remains unclear. Here we show that chemotherapeutic agent cisplatin promotes glycolysis, which is suppressed by *PFKFB3* deletion. Mechanistically, cisplatin induces PFKFB3 acetylation at lysine 472 (K472), which impairs activity of the nuclear localization signal (NLS) and accumulates PFKFB3 in the cytoplasm. Cytoplasmic accumulation of PFKFB3 facilitates its phosphorylation by AMPK, leading to PFKFB3 activation and enhanced glycolysis. Inhibition of PFKFB3 sensitizes tumor to cisplatin treatment in a xenograft model. Our findings reveal a mechanism for cells to stimulate glycolysis to protect from DNA damage and potentially suggest a therapeutic strategy to sensitize tumor cells to genotoxic agents by targeting PFKFB3.

## Abstract (web summary)

Enhanced glycolysis in cancer cells has been associated with protection from DNA damage. Here the authors show that DNA damaging signals induce acetylation of PFKFB3 at lysine K472 and promote its cytosolic accumulation, which enhances glycolysis, resulting in protection from cisplatin-induced cell death.

## Introduction

Rapidly proliferating cells such as cancer cells have increased glucose uptake, enhanced glycolysis and reduced oxygen consumption even in the presence of normal oxygen supply, leading to the accumulation of lactate. This phenomenon, commonly referred to as the Warburg effect^1,2^, is interpreted as a need to meet the demand by actively dividing cells for glycolytic and Krebs cycle intermediates to support biosynthesis. The 6-phosphofructo-1-kinase 1 (PFK-1) catalyzes the first irreversible reaction (committed step) of glycolysis, converting fructose-6-phosphate (F6P) to fructose-1,6-bisphosphate (F1,6BP). As a result, PFK-1 serves as the focal point for the integration of multiple signals, including notably allosteric regulation by adenosine triphosphate (ATP) and adenosine monophosphate (AMP) to sense intracellular energy level and activation by fructose-2,6-bisphosphate (F2,6BP) in response to the change in blood glucose^3^. F2,6BP is controlled by phosphofructokinase-2/fructose-2,6-bisphosphatases (PFKFBs), a family of bifunctional enzyme that contains a kinase and a phosphatase domain and catalyzes the synthesis (phosphorylation) of F2,6BP from and degradation (dephosphorylation) of F2,6BP to fructose-6-bisphosphate (F6P). F2,6BP can override the ATP inhibition of PFK-1, making PFKFB as a critical key enzyme in the control the rate of glycolysis. Human genome encodes four PFKFB isoenzymes, of which PFKFB3 has two unique properties. It has a much higher kinase/phosphatase activity ratio (710-fold) while the other PFKFBs have similar kinase and phosphatase activity^4^. This makes PFKFB3 function mainly in producing F2,6BP and promoting glycolytic flux^5^. Additionally, unlike the other three PFKFBs which all localize predominantly in the cytoplasm where the glycolysis occurs, PFKFB3 is mainly localized in the nucleus^6^. The significance of nuclear localization of PFKFB3 remains elusive.

PFKFB3 has been reported to play important roles in promoting tumor cell growth. Inhibition of PFKFB3 by chemical inhibitors or genetic silence dramatically reduces glycolytic flux, Ras-driven transformation and tumor growth in athymic mice^7–9^. Furthermore, inhibition of PFKFB3 impairs pathological angiogenesis and induces tumor vessel normalization, leading to reduced metastasis and improved chemotherapy^10–12^. It was also recently reported that PFKFB3 promotes breast cancer cell survival during microtubule poison-induced mitotic arrest^13^. It is currently unclear how the activity of PFKFB3 is stimulated to facilitate tumor growth and survival.

PFKFB3 level is regulated at both transcriptional level and by protein stability. It is transcriptionally stimulated by lipopolysaccharide and hypoxia^7,14^, and its protein stability is controlled by the E3 ubiquitin ligases APC/C-Cdh1 and SCF/CRL1^β-TrCP^ during the cell cycle^15–17^. In addition to the regulation of protein level, PFKFB3 activity is also known to be regulated by post-translational modifications. Under energy crisis, PFKFB3 is phosphorylated by AMP-activated kinase (AMPK) at S461 residue and this phosphorylation increases PFKFB3 activity to stimulate glycolysis and ATP production^18,19^. PFKFB3 was also found to be di-methylated at arginine 131/134 residues. Carbon monoxide reduces methylation of PFKFB3 and promotes its degradation through proteasome pathway, thus shunting glucose usage from glycolysis to the pentose phosphate pathway for NADPH generation^20^. In this study, we demonstrate that PFKFB3 has a key role in protecting cancer cells from apoptosis induced by chemotherapy agent. We found that DNA damage agents stimulate PFKFB3 acetylation at lysine 472 (K472) to increase PFKFB3 cytoplasmic accumulation and ability to promote glycolysis, which is important for cell survival in response to DNA damaging chemotherapeutic agents. We also show that inhibition of PFKFB3 sensitize cells to cisplatin-induced apoptosis. Our observations uncover a novel mechanism of PFKFB3 regulation by acetylation-mediated cytoplasmic accumulation and suggest a potential therapeutic strategy of anticancer chemotherapy through targeting PFKFB3.

## Results

### Inhibition of PFKFB3 promotes cisplatin-induced apoptosis

**Fig. 1:**
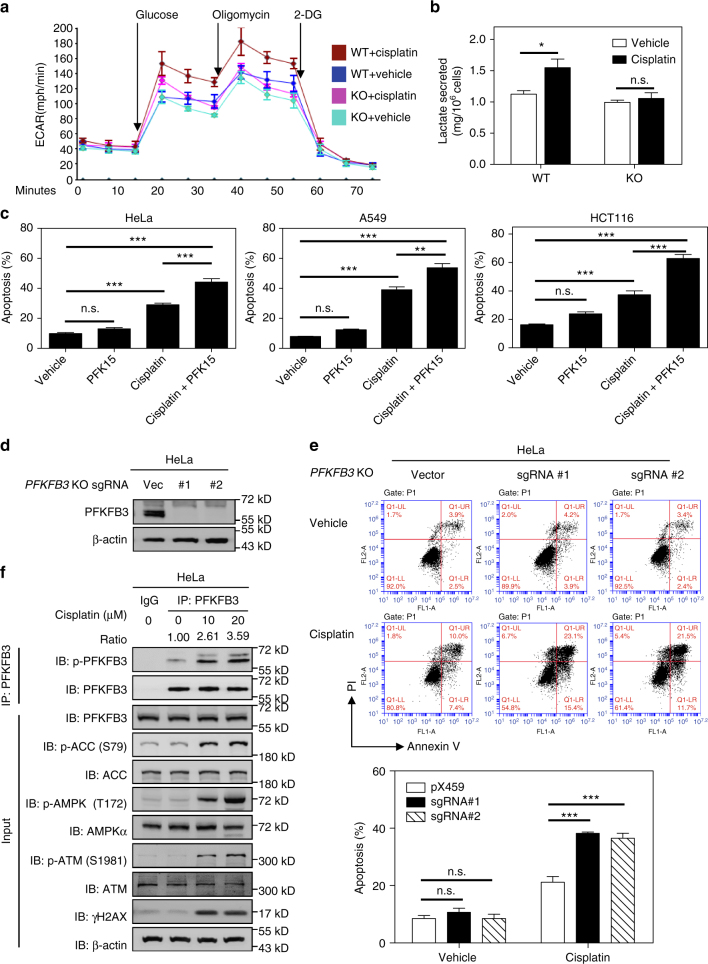
Inhibition of PFKFB3 cooperates with cisplatin to promote cancer cell apoptosis. **a** ECAR assay was performed in WT or *PFKFB3* KO HeLa cells treated with or without cisplatin (10 μM) for 12 h using a Seahorse Bioscience XFe 96 analyzer system. Data are shown as mean with s.d. of three biological replicates. KO, knockout. **b** Lactate secretion in the culture medium of WT or *PFKFB3* KO HeLa cells treated with or without cisplatin (10 μM) for 24 h. Data are presented as mean ± s.d. of three biological replicates, and statistical analyses were performed using two-way ANOVA with Bonferroni’s post-test. *P < 0.05; n.s., not significant. **c** Combined use of cisplatin and PKF15 efficiently induced apoptosis of cancer cells. HeLa, A549 and HCT116 cells were treated with PFK15 (10 μM), cisplatin (10 μM for HeLa, 30 μM for A549 and 50 μM for HCT116 cells) or the combination of the both agents for 24 h. Cell apoptosis was assessed by flow cytometry analysis of Annexin V and/or PI-positive cells. Data are presented as mean ± s.d. of three biological replicates, and statistical analyses were performed by using one-way ANOVA with Bonferroni’s post-test. **p < 0.01 and ***p < 0.001 for the indicated comparison; n.s., not significant. **d** Verification of *PFKFB3* knockout in HeLa cells used in **d** by immunoblotting. *PFKFB3* knockout cells were generated by the CRISPR/Cas9 gene editing system. **e** Loss of *PFKFB3* promotes cisplatin-induced apoptosis. WT (pX459) or *PFKFB3* KO (sgRNA-1 and -2) HeLa cells were exposed to cisplatin (10 μM) for 24 h. Cell apoptosis was assessed by flow cytometry analysis of Annexin V and/or PI-positive cells. Data are presented as mean ± s.d. of three biological replicates, and statistical analyses were performed by using two-way ANOVA with Dunnett’s post-test. ***P < 0.001 for the indicated comparison; n.s., not significant. **f** Cisplatin treatment enhanced phosphorylation of PFKFB3. HeLa cells were treated with cisplatin at indicated concentrations for 24 h. Endogenous PFKFB3 protein was immunoprecipitated from HeLa cells. Relative PFKFB3 phosphorylation was normalized by PFKFB3 protein

Cisplatin is a widely used chemotherapy drug in the treatment of many solid tumors, such as lung, cervix, ovarian, bladder, testicular and head and neck cancer^21^. Cisplatin treatment results in DNA damage-triggered cell-cycle arrest and apoptosis^22,23^. Reprogramming energy metabolism is a hallmark of cancer. Rapidly proliferating cancer cells show enhanced glycolysis for ATP production even in the presence of normal oxygen supply. We measured extracellular acidification rate (ECAR) and lactate secretion, and found that cisplatin treatment promoted glycolysis rate of HeLa cells (Fig. 1a, b). In addition, we also observed that endogenous 6-phosphofructokinase activity is increased (Supplementary Fig. 1a). This led us to explore the role of PFKFB3, the most ubiquitous PFKFB family isoenzyme and a potent glycolysis stimulator, in the process of cisplatin-induced apoptosis. We tested the combined effect of cisplatin treatment and inhibition of PFKFB3 by PFK15 [1-(4-pyridinyl)-3-(2-quinolinyl)-2-propen-1-one^24^], a potent PFKFB3 inhibitor that can dramatically reduce ATP generation in cells (Supplementary Fig. 1b). HeLa, A549 and HCT116 cells were treated with cisplatin in the presence or absence of PFK15. We found that PFK15 alone did not significantly affect apoptosis, but significantly enhanced apoptosis induced by cisplatin (Fig. 1c). To further confirm the role of PFKFB3 inhibition in enhancing cisplatin-induced cell apoptosis, we generated *PFKFB3* knockout HeLa cells using the CRISPR/Cas9 technology. Two independent *PFKFB3* knockout clones were obtained with two independent guide sequences and were verified by immunoblotting and DNA sequencing (Fig. 1d and Supplementary Fig. 1c). We found that *PFKFB3* deletion also sensitized HeLa cells to apoptosis induced by cisplatin (Fig. 1e). Importantly, deletion of *PFKFB3* blocked the activation of glycolysis in cells treated with cisplatin (Fig. 1a, b), suggesting a potential role of enhanced glycolysis in cell protection.

Phosphorylation of PFKFB3 at S461 by AMPK is a mechanism known to activate PFKFB3 and promote glycolysis^18,19^. We asked if cisplatin treatment might affect S461 phosphorylation of PFKFB3. The phosphorylation of S461 in the presence or absence of cisplatin was measured by an antibody recognizing phospho-AMPK substrate motif [LXRXX(pS/pT)]. A substantial increase of endogenous S461 phosphorylation was observed upon cisplatin treatment (Fig. 1f). These data indicate cisplatin treatment activates PFKFB3 by increasing S461 phosphorylation and thereby promoting glycolysis, which is important for cell survival in response to cisplatin.

### PFKFB3 is acetylated at K472

**Fig. 2:**
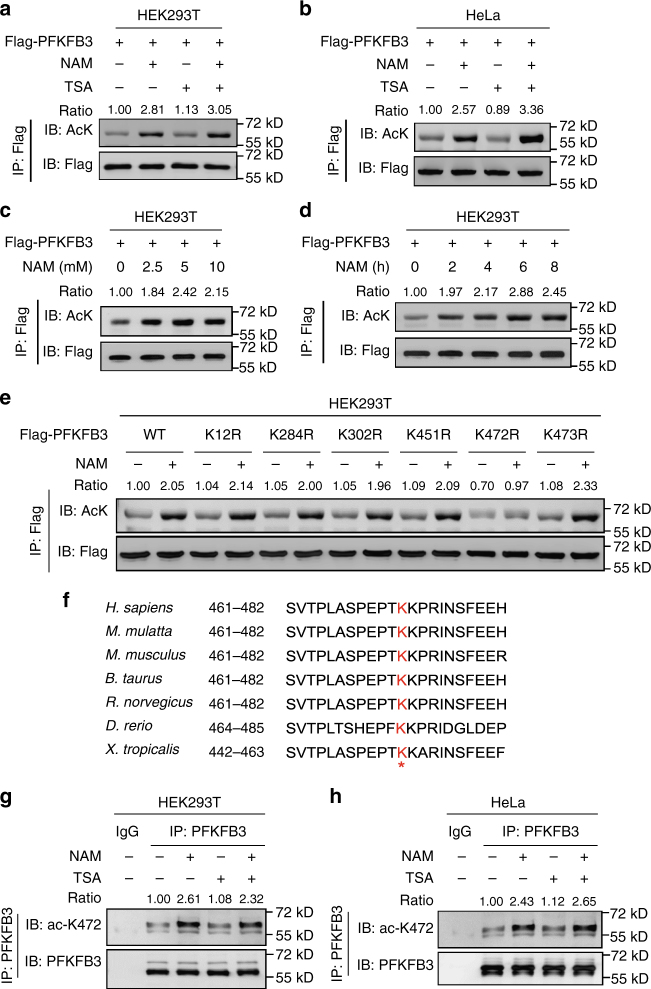
PFKFB3 is acetylated at K472.** a**,** b** Acetylation of PFKFB3 was increased by deacetylase inhibitors. Flag-tagged PFKFB3 was ectopically expressed in **a** HEK293T and **b** HeLa cells. Cells were treated with NAM, TSA or both for the duration indicated. Flag-PFKFB3 was immunoprecipitated from cell lysate and its acetylation was examined with a pan-acetylated lysine antibody (α-AcK). Relative PFKFB3 acetylation was normalized by Flag protein. **c**,** d** Acetylation level of PFKFB3 was increased by NAM treatment in a dose- and time-dependent manner. Flag-tagged PFKFB3 was ectopically expressed in HEK293T cells. Cells were treated with NAM at the indicated concentrations for 6 h (**c**), or treated with 5 μM NAM for the duration indicated (**d**). Flag-PFKFB3 was immunoprecipitated and its acetylation was examined with α-AcK. **e** K472 is the major acetylation residue of PFKFB3. Flag-tagged WT PFKFB3 or mutants (K12R, K284R, K302R, K451R, K472R, and K473R) were expressed in HEK293T cells, followed by treatments with or without 5 μM NAM. Flag-PFKFB3 was immunoprecipitated and its acetylation was examined with α-AcK. **f** K472 in PFKFB3 is evolutionarily conserved in vertebrates. Alignment of the sequences around PFKFB3 K472 from different species is shown. **g**,** h** Endogenous PFKFB3 is acetylated at K472. Endogenous PFKFB3 protein was purified from HEK293T or HeLa cells after NAM and TSA treatment as indicated. Acetylation of K472 was examined with an antibody specifically recognizing the acetylated K472 residue of PFKFB3 (α-acK472). Relative PFKFB3 K472 acetylation was normalized by PFKFB3 protein. Data are representative of three independent experiments

That inhibition of PFKFB3 enhanced the cell apoptosis induced by cisplatin intrigued us to explore the mechanism of PFKFB3 regulation by DNA damaging agents. Our previous studies have demonstrated that acetylation of metabolic enzymes is an important mechanism of metabolic modulation^25,26^. We performed mass spectrometry analysis, which demonstrated that PFKFB3 may be acetylated at multiple lysine residues (Supplementary Fig. 2a). To confirm its acetylation, Flag-tagged PFKFB3 was ectopically expressed in HEK293T and HeLa cells and its acetylation level was tested by a pan-anti-acetylated lysine antibody. The result demonstrated that ectopically expressed PFKFB3 was indeed acetylated and its acetylation was significantly enhanced after treatment with nicotinamide (NAM), an inhibitor of the SIRT family deacetylases^27–29^, but not trichostatin A (TSA), an inhibitor of histone deacetylase I and II^30,31^ (Fig. 2a, b). The acetylation level of PFKFB3 was increased by NAM treatment in a dose- and time-dependent manner (Fig. 2c, d).

Mass spectrometric analysis suggested six putative acetylation lysine (K) residues in PFKFB3, including K12, K284, K302, K451, K472 and K473 (Supplementary Fig. 2a). To determine which lysine residue(s) is the major acetylation site(s), we mutated individually each of the six putative lysine residues to arginine (R). The replacement of lysine with arginine retains the positive charge and is often used as a deacetylation mimetic mutant. Among the six mutants, the K472R substantially reduced the acetylation of PFKFB3 (Fig. 2e), suggesting that K472 is the major acetylation site of PFKFB3 (Supplementary Fig. 2b).

Protein sequence alignment showed that K472 is evolutionarily conserved in vertebrate PFKFB3 (Fig. 2f). To confirm K472 acetylation in vivo, we generated an antibody (ac-K472) using the K472-acetylated peptide of PFKFB3. Dot blot assay showed that the ac-K472 antibody preferentially detected the acetylated peptide, but not the unmodified peptide (Supplementary Fig. 2c), demonstrating the specificity of this antibody. Importantly, a substantial increase of endogenous PFKFB3 K472 acetylation was detected by the ac-K472 antibody when the HEK293T (Fig. 2g) and HeLa (Fig. 2h) cells were treated with NAM, while TSA treatment had no effect on PFKFB3 K472 acetylation. These data show that K472 acetylation of PFKFB3 occurs under physiological conditions and is deacetylated by member(s) of the SIRT family of deacetylase.

### Acetylation mimetic mutant of PFKFB3 disrupts its NLS motif

**Fig. 3:**
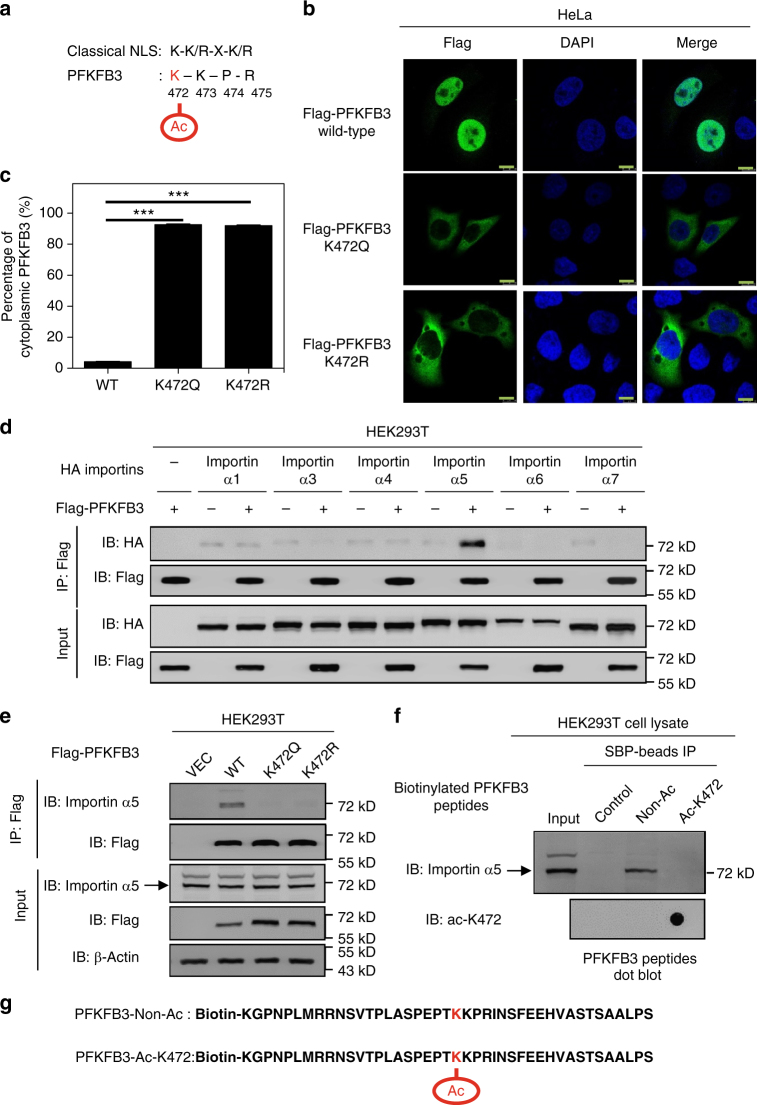
Acetylation mimetic mutant of PFKFB3 disrupts its NLS motif. **a** K472 is located as the first lysine of a putative NLS motif. Schematic representation of the consensus sequence K(K/R)X(R/K) of a classical nuclear localization signal is shown. **b** K472 mutation results in cytoplasmic accumulation of PFKFB3. Flag-tagged WT, K472Q or K472R mutant of PFKFB3 was transfected into HeLa cells. After 24 h, cells were fixed with 4% paraformaldehyde, followed by incubation with anti-Flag primary antibody and then with Alexa Fluor 488-labeled secondary antibody (green). DAPI (blue) was used for nuclei staining. Scale bars: 10 μm. **c** Quantification of percentage of cytoplasmic PFKFB3 described in **b**. At least 50 cells were randomly selected and quantified for each group. Data are presented as mean ± s.d., and statistical analyses were performed using one-way ANOVA with Dunnett’s post-test. ***P < 0.001 for the indicated comparison. **d** PFKFB3 specifically binds to importin α5. Flag-tagged PFKFB3 was co-expressed with different HA-tagged importin α subunits as indicated in HEK293T cells. PFKFB3 was immunoprecipitated with Flag beads, and importin α subunits that associate with it were detected with an HA antibody. **e** K472 mutation of PFKFB3 abolishes its interaction with importin α5. Flag-tagged WT, K472Q or K472R mutant of PFKFB3 was transfected into HEK293T cells. Flag-PFKFB3 was immunoprecipitated with Flag beads and its associated endogenous importin 5α was detected by immunoblotting. **f** K472-acetylated peptide loses binding affinity to importin 5α. Biotinylated PFKFB3 peptides containing either acetylated or non-acetylated K472 residue were synthesized and incubated with HEK293T cell lysate. Peptides were then purified with streptavidin sepharose, followed by immunoblotting with an importin 5α antibody. Peptides were verified by dot blotting with the α-acK472 antibody.** g** Schematic representation of the biotinylated peptides used in **e**. The K472 residue is highlighted. Data are representative of at least two independent experiments

Unlike other PFKFB enzymes that are localized in the cytoplasm, PFKFB3 is mainly localized in the nucleus, though the reason remains unclear^6^. PFKFB3 contains a classical nuclear localization signal (NLS) as “KKPR” (amino acids 472–475, Fig. 3a)^6^. Since acetylation neutralizes the positive charge of lysine residue, we asked if K472 acetylation may interfere with nuclear localization of PFKFB3. To this end, we mutated K472 to glutamine (K472Q) or arginine (K472R) and determined their subcellular localization. The replacement of lysine with glutamine abolishes the positive charge and often mimic acetylation^32^. Epitope tagged PFKFB3 wild-type (WT), K472Q or K472R mutant was expressed in HeLa cells and the subcellular localization was examined by immunofluorescence (IF). In contrast to the nuclear localization of WT PFKFB3, the acetylation mimetic mutant K472Q was mainly localized in cytoplasm, indicating that acetylation of K472 may disrupt PFKFB3-NLS motif (Fig. 3b, c). Surprisingly, the K472R mutant also showed cytoplasmic distribution (Fig. 3b, c). This might be due to the fact that the first lysine residue of a given NLS is critical for its recognition by the importin complex^33,34^. Therefore, substitution of the first lysine with any other amino acid may abolish NLS function, which is supported by observations from a previous study^35^. Moreover, similar results were observed in U2OS cells that WT GFP-PFKFB3 was nuclear whereas the K472Q or K472R mutant was cytoplasmic (Supplementary Fig. 3a).

To gain mechanistic insights of PFKFB3 nuclear localization, we searched for importin(s) that is responsible for transportation of PFKFB3 into nucleus. Immunoprecipitation assay showed that PFKFB3 specifically binds to the importin α5 (Fig. 3d), but not other importin α subunits. Importantly, PFKFB3 with mutation of K472Q or K472R abolished its interaction with importin α5 (Fig. 3e), supporting a model that K472 acetylation or mutation in PFKFB3 impairs its NLS function. To further demonstrate whether the interaction of PFKFB3 with importin α5 is regulated by K472 acetylation, we synthesized PFKFB3 peptides containing either acetylated or non-acetylated K472 residue. The peptides were biotinylated, conjugated with streptavidin beads and used for importin interaction assays. After incubation with HEK293T cell lysate, only the K472-unacetylated peptide, but not the K472-acetylated peptide, pulled down endogenous importin α5 (Fig. 3f, g), supporting a direct role of K472 acetylation in modulating PFKFB3 interaction with importin α5.

Three-dimensional structure of human importin α5 with bound PFKFB3-NLS is currently not available. We thus analyzed the available data of crystal structure of mouse importin α1 bound with the classic NLS peptide^36^. Notably, the first lysine of NLS physically interacts with importin α subunit. Consistent with our previous data, structure modeling analyses showed that acetylation of the first lysine in the NLS effectively interferes its interaction with importin α subunit by neutralizing the positive charge of side chain and introducing steric hindrance (Supplementary Fig. 3b). Together, these results indicate that acetylation of lysine K472 within the NLS of PFKFB3 prevents its recognition by the importin complex, offering a molecular mechanism for acetylation-dependent regulation of PFKFB3 cytoplasmic nuclear localization.

### Cytoplasmic PFKFB3 potentiates S461 phosphorylation

**Fig. 4:**
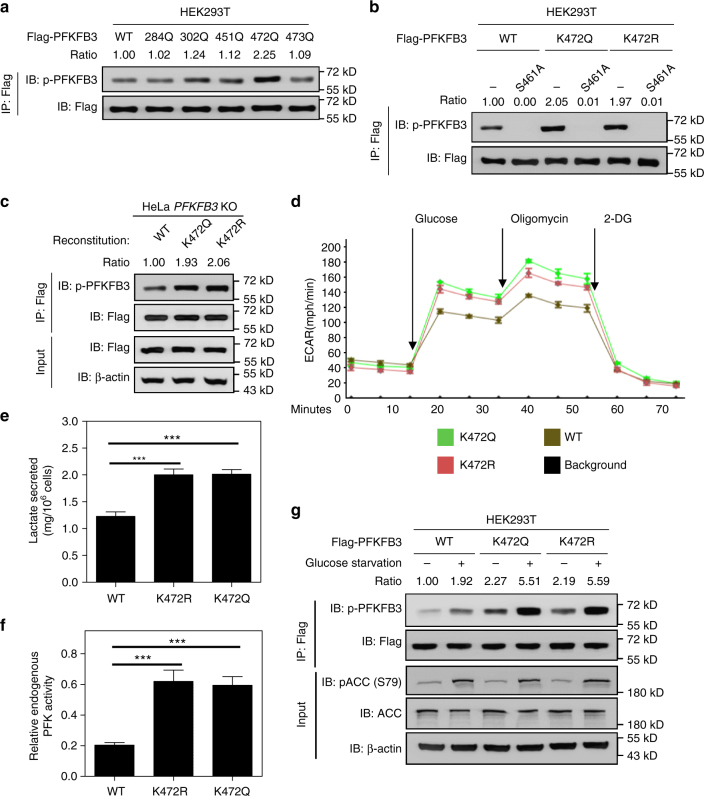
Cytoplasmic PFKFB3 potentiates S461 phosphorylation. **a** K472Q mutant of PFKFB3 shows higher phosphorylation by AMPK. Flag-tagged WT PFKFB3 or mutants were transiently expressed in HEK293T cells. PFKFB3 was immunoprecipitated with Flag beads, followed by immunoblotting with the antibody recognizing phospho-AMPK substrate motif. Relative PFKFB3 phosphorylation was normalized by Flag protein. **b** K472Q or K472R mutation enhances PFKFB3 phosphorylation by AMPK, which is blocked by S461A mutation. Flag-tagged WT PFKFB3 or its mutants were transiently expressed in HEK293T cells. Phosphorylation of Flag-tagged proteins was examined as in **a**. **c** Establishment of *PFKFB3* KO HeLa cells that stably express Flag-tagged WT, K472Q or K472R mutant of PFKFB3. **d** HeLa cells expressing K472 mutants exhibit increased glycolysis (ECAR rate) compared to those expressing WT PFKFB3. HeLa stable cells described in **c** were subjected to ECAR assay. Data are shown as mean with s.d. of three biological replicates. **e** HeLa cells expressing K472 mutants exhibit increased lactate secretion compared to those expressing WT PFKFB3. Lactate in the culture medium of *PFKFB3* KO HeLa cells that stably express Flag-tagged WT, K472Q or K472R mutant of PFKFB3 were measured. Data are presented as mean ± s.d. of three biological replicates, and statistical analyses were performed using one-way ANOVA with Dunnett’s post-test. ***P < 0.001 for the indicated comparison; n.s., not significant. **f** HeLa cells expressing K472 mutants exhibit increased endogenous PFK activity compared to those expressing WT PFKFB3. Endogenous PFK activity of *PFKFB3* KO HeLa cells that stably express Flag-tagged WT, K472Q or K472R mutant of PFKFB3 were assayed. Data are presented as mean ± s.d. of three biological replicates, statistical analyses were performed using one-way ANOVA with Dunnett’s post-test. ***P < 0.001 for the indicated comparison; n.s., not significant. **g** Glucose-deprivation-induced S461 phosphorylation of PFKFB3 was exaggerated by K472Q or K472R mutation. Flag-tagged WT, K472Q or K472R mutant of PFKFB3 were expressed in HEK293T cells. Cells were cultured in medium containing 25 μM or 0 μM glucose for 5 h. Data are representative of at least two independent experiments

The functional significance of PFKFB3 subcellular localization may be reflected by the fact that the cytoplasmic PFKFB3 is more potent in stimulating glycolysis that occurs in cytosol^6^. Phosphorylation of PFKFB3 at S461 by AMPK is a mechanism that is known to activate PFKFB3 and promote glycolysis^18^. We asked if cytoplasmic localization affects PFKFB3 S461 phosphorylation. Therefore, PFKFB3 S461 phosphorylation was examined with a phospho-specific antibody. We found that K472Q mutant showed significantly higher S461 phosphorylation when compared with the WT PFKFB3 (Fig. 4a). The increase of S461 phosphorylation was abolished by Lambda phosphatase treatment (Supplementary Fig. 4) and S461A mutation (Fig. 4b), confirming the specificity of the phospho-antibody. Additionally, the K472R mutant, which is cytoplasmic, also displayed higher S461 phosphorylation than the WT PFKFB3 (Fig. 4b and Supplementary Fig. 4). Collectively, these results suggest that K472 acetylation increases PFKFB3 cytoplasmic localization and S461 phosphorylation, leading to increased enzymatic activity.

To test the physiological significance of this regulation, we stably expressed WT, K472Q or K472R mutant of PFKFB3 in *PFKFB3* knockout HeLa cells. Consistent with its role in promoting glycolysis, K472Q or K472R mutant cells, which have higher S461 phosphorylation and activity of PFKFB3, had elevated ECAR rate and lactate secretion when compared to cells expressing WT PFKFB3 (Fig. 4c–e). Meanwhile, the activity of endogenous 6-phosphofructokinase in K472Q or K472R mutant cells was also elevated (Fig. 4f). It is well known that energy stress such as glucose starvation activates AMPK and induces PFKFB3 S461 phosphorylation. Interestingly, the effect of glucose starvation on S461 phosphorylation was markedly exaggerated by either K472Q or K472R mutation (Fig. 4g), suggesting that cytoplasmic localization makes PFKFB3 more sensitive to energy stress-induced phosphorylation and activation.

### SIRT1 is a major deacetylase for PFKFB3

**Fig. 5:**
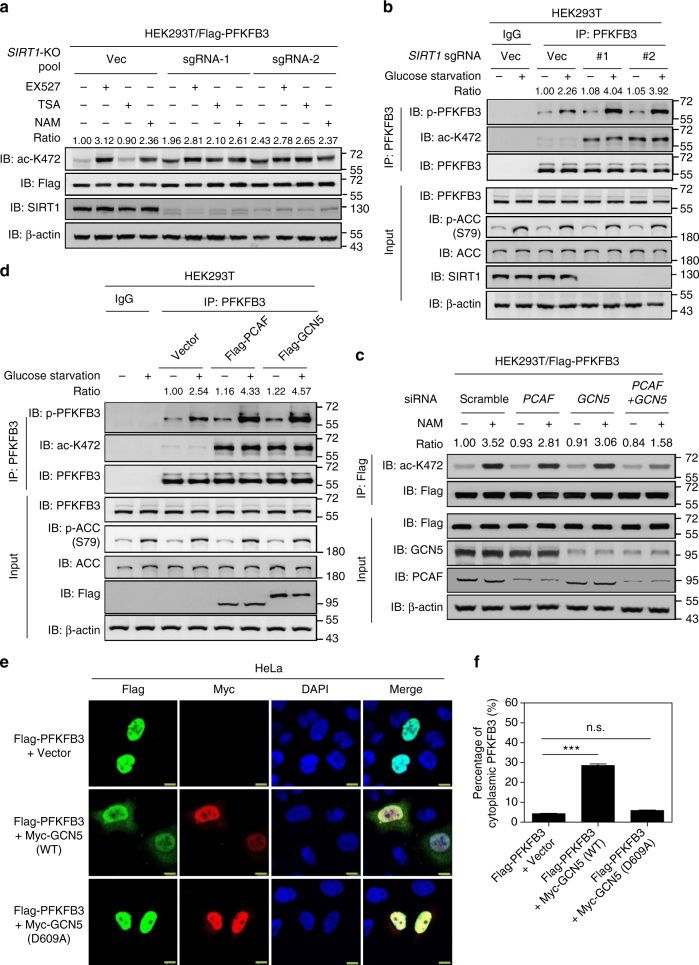
SIRT1 and PCAF/GCN5 are potential deacetylase and acetyltransferases for K472 of PFKFB3 respectively. **a** Loss of *SIRT1* resulted in increased K472 acetylation of PFKFB3. Flag-tagged PFKFB3 was expressed in HEK293T WT or *SIRT1* knockout cell pools generated by using the CRISPR/Cas9 gene editing system. Cells were then treated with EX527 (10 μM), NAM (5 μM) or TSA (0.5 μM) for 8 h. K472 acetylation of Flag-tagged PFKFB3 was determined by immunoblotting. **b** *SIRT1* deletion enhanced PFKFB3 S461 phosphorylation induced by glucose starvation. HEK293T WT or SIRT1 knockout cells were treated with 25 μM glucose or 0 μM glucose for 5 h. Endogenous PFKFB3 protein was immunoprecipitated. Immunoblotting was performed with the indicated antibodies. Relative PFKFB3 phosphorylation was normalized by PFKFB3 protein. **c** Combined knockdown of *PCAF* and *GCN5* abolishes NAM-induced PFKFB3 K472 acetylation. Flag-tagged PFKFB3 was co-expressed with siRNAs targeting *PCAF*, *GCN5* or both in HEK293T cells. Cells were then treated with NAM (5 μM) for 6 h. K472 acetylation levels of purified Flag-PFKFB3 proteins were determined with α-acK472 antibody. **d** Ectopic expression of PCAF or GCN5 enhanced glucose-deprivation induced S461 phosphorylation of PFKFB3. HEK293T cells were transfected with empty vector, PCAF or GCN5 for 36 h, followed by treatment with 25 μM glucose or 0 μM glucose for 5 h. Endogenous PFKFB3 protein was immunoprecipitated with PFKFB3 antibody. Immunoblotting was performed with the indicated antibodies. Relative PFKFB3 phosphorylation was normalized by PFKFB3 protein. **e** Ectopic expression of GCN5-induced cytosolic retention of PFKFB3. HeLa cells were co-transfected with Flag-tag PFKFB3 and Myc-tag WT or acetyltransferase activity-dead mutant of GCN5 (D609A). Localization of PFKFB3 and expression of GCN5 were examined by IF staining with Flag and Myc antibodies, respectively. DAPI (blue) was used for nuclei staining. Scale bars: 10 μm. **f** Quantification of cytoplasmic percentage of PFKFB3 described in **e**. At least 100 cells were randomly selected and quantified for each group. Data are presented as mean ± s.d., and statistical analyses were performed using one-way ANOVA with Dunnett’s post-test. ***P < 0.001 for the indicated comparison; n.s., not significant. Data are representative of at least two independent experiments

Our earlier observation that NAM increases PFKFB3 acetylation (Fig. 2a) suggests a possible involvement of NAD^+^-dependent Sirtuins (SIRTs) in PFKFB3 deacetylation. Mammalian SIRT1–3 display robust deacetylation activity, while SIRT4–7 have no detectable or weak deacetylase activity and show activity towards other types of lysine modifications^37,38^. Given that SIRT3 is localized in the mitochondria^39^, we examined whether SIRT1 or SIRT2 could be deacetylase for PFKFB3. Ectopically expression of SIRT1, but not SIRT2, suppressed the acetylation of PFKFB3 (Supplementary Fig. 5a), indicating SIRT1 may be the major deacetylase for PFKFB3. Moreover, a physical interaction was observed between PFKFB3 and SIRT1, but not SIRT2 (Supplementary Fig. 5b). In addition, EX527, a potent and selective SIRT1 inhibitor^40^, increased K472 acetylation as efficient as NAM (Fig. 5a).

To further confirm SIRT1 is a physiological deacetylase for PFKFB3, we generated *SIRT1* knockout pools in HEK293T cell line using the CRISPR/Cas9 gene editing system. Loss of *SIRT1* resulted in a dramatic increase of K472 acetylation of PFKFB3, which could no longer be enhanced by EX527 or NAM treatment (Fig. 5a). We further isolated two independent and single-cell-derived *SIRT1* knockout clones (Supplementary Fig. 5c). K472 acetylation of endogenous PFKFB3 was significantly elevated in the *SIRT1* knockout cells (Supplementary Fig. 5d). These data demonstrate that SIRT1 is the major deacetylase for PFKFB3. Interestingly, glucose starvation-induced PFKFB3 S461 phosphorylation was further enhanced by *SIRT1* deletion (Fig. 5b). This indicates that PFKFB3 acetylation promotes its phosphorylation by AMPK, possibly through suppressing PFKFB3 nuclear transportation.

### PCAF and GCN5 are major acetyltransferases for PFKFB3

To identify the acetyltransferase responsible for PFKFB3 acetylation at K472, we examined five acetyltransferases, p300 (E1A binding protein, 300 kDa), CBP (CREB binding protein), PCAF (p300/CBP-associated factor, also known as lysine acetyltransferase 2B, KAT2B), GCN5 (also known as KAT2A) and TIP60 (also known as KAT5). Ectopically expression of p300, CBP, PCAF and GCN5 increased PFKFB3 acetylation as detected by a pan-acetylated lysine antibody (Supplementary Fig. S6a). However, only PCAF and GCN5, both of which belong to the Gcn5 N-acetyltransferase (GNAT) subfamily^41^, significantly increased PFKFB3 K472 acetylation as detected by the acetyl K472 antibody (Supplementary Fig. 6a). In addition, PCAF and GCN5 failed to increase acetylation of the PFKFB3 K472R mutant (Supplementary Fig. 6b), indicating that PCAF and GNC5 specifically acetylate K472 in PFKFB3. Consistent with these observations, a combined knockdown of *PCAF* and *GCN5* by small interfering RNA (siRNA) abolished NAM-induced PFKFB3 K472 acetylation (Fig. 5c), further supporting the notion that PCAF and GCN5 are the major cellular acetyltransferases for PFKFB3 K472. Moreover, ectopically expression of PCAF or GCN5 also significantly increased endogenous PFKFB3 K472 acetylation, and enhanced PFKFB3 S461 phosphorylation under glucose starvation (Fig. 5d), supporting a model that PCAF and GCN5 induce PFKFB3 K472 acetylation to promote its S461 phosphorylation and activity.

### GCN5 promotes cytosolic retention of PFKFB3

The data above suggest a model that PFKFB3 acetylation leads to its retention in the cytoplasm, which facilitates its phosphorylation and activation by AMPK. To further support this model, we first quantified the acetylation level of PFKFB3 K472 residue in response to different treatments, such as NAM treatment and co-expression of PCAF/GCN5. Our results showed that co-expression with GCN5 or PCAF induced K472 acetylation of about 40% of total PFKFB3. NAM treatment resulted in about 10% PFKFB3 acetylation (Supplementary Fig. 7a-c), indicating a significant fraction of endogenous PFKFB3 could be acetylated. We next determined PFKFB3 subcellular localization with co-expression of GCN5, and found GCN5 expression induced evident PFKFB3 retention in the cytoplasm, while the acetyltransferase activity-dead mutant of GCN5 (D609A)^42^ failed to do this (Fig. 5e, f). Quantification of IF images demonstrated that GCN5 expression resulted in approximately 30% of PFKFB3 accumulation in the cytoplasm, a ratio similar to that of acetylated PFKFB3 induced by GCN5 expression. This process was accompanied by an elevation of endogenous 6-phosphofructokinase activity (Supplementary Fig. 6c). Meanwhile, co-expression of GCN5 largely reduced the interaction between PFKFB3 and importin α5 in a dose-dependent manner (Supplementary Fig. 6d). These results further support that K472 acetylation of PFKFB3 disrupts its interaction with importin α5 and induces cytoplasmic localization.

### Cisplatin induces acetylation and phosphorylation of PFKFB3

**Fig. 6:**
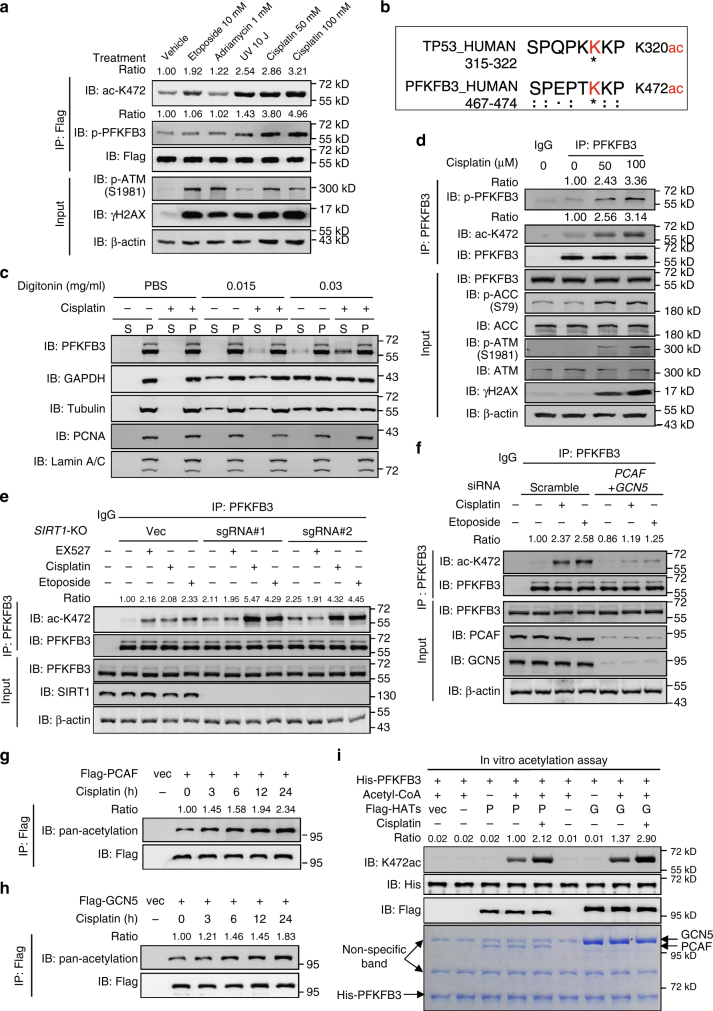
Cisplatin induces K472 acetylation and S461 phosphorylation of PFKFB3. **a** DNA damage signals induced PFKFB3 K472 acetylation. Flag-tagged PFKFB3 was expressed in HEK293T cells, which were then treated with etoposide (10 μM), adriamycin (1 μM), UV irradiation (10 J/m^2^) and cisplatin (50 or 100 μM) for 24 h. Flag-PFKFB3 was immunoprecipitated with Flag beads and immunoblotting was performed with the antibodies indicated. Relative PFKFB3 K472 acetylation and phosphorylation were normalized by Flag protein. **b** The amino acid sequence near K472 of PFKFB3 displays high similarity with the sequence near K320 of TP53. **c** Cisplatin treatment induces PFKFB3 cytoplasmic accumulation. HEK293T cells were treated with or without cisplatin (50 μM) for 24 h before harvest. Cells were then suspended in PBS and treated with a gradient concentration of digitonin. Supernatant and precipitate were collected for immunoblotting with indicated antibodies. S, supernatant; P, precipitate. **d** Cisplatin induces K472 acetylation and S461 phosphorylation of endogenous PFKFB3. Endogenous PFKFB3 protein was purified from HEK293T cells after cisplatin treatment as indicated for 24 h. **e** Cisplatin or etoposide treatment enhances K472 acetylation in *SIRT1* knockout cells. Endogenous PFKFB3 protein were purified from WT or *SIRT1* knockout HEK293T cells treated with EX527 (10 μM), cisplatin (50 μM) or etoposide (10 μM) for 24 h. **f** Combined knockdown of *PCAF* and *GCN5* abolishes cisplatin- or etoposide-induced PFKFB3 K472 acetylation. HEK293T cells were transfected with siRNAs targeting PCAF and GCN5. After 60 h, cells were treated with cisplatin (50 μM) or etoposide (10 μM) for 24 h. **g**, **h** Cisplatin treatment induces pan-acetylation of PCAF and GCN5. Flag-tagged PCAF or GCN5 was expressed in HEK293T cells, which were treated with cisplatin for the duration indicated at a concentration of 50 μM. Relative pan-acetylation level of PCAF or GCN5 was normalized by Flag protein. **i** Cisplatin increases acetyltransferase activity of PCAF and GCN5. Flag-tag PCAF or GCN5 was purified from HEK293T cells treated with or without cisplatin (50 μM) for 24 h, then incubated with recombinant His-PFKFB3 in acetylation assay buffer. Purified proteins visualized by Coomassie blue staining are shown (lower panel). Data are representative of at least two independent experiments

To determine the physiological significance of PFKFB3 K472 acetylation, we investigated if the K472 acetylation is dynamically regulated in vivo. Because PFKFB3 is a metabolic enzyme involved in the glycolytic pathway, we first tested if its K472 acetylation is regulated by glucose level. However, K472 acetylation was not sensitive to glucose concentrations in the medium (Supplementary Fig. 8a). Additionally, K472 acetylation was not changed by glutamine level, serum level, insulin stimulation or oxidative stress condition either (data not show). Given our observation that PFKFB3 inhibition sensitizes cells to cisplatin, we speculated that a nuclear stress may affect PFKFB3 acetylation and signal to cytoplasmic glycolysis. To test this possibility, HEK293T cells were treated with several DNA damage signals, including ultraviolet (UV) irradiation and three chemotherapy drugs, etoposide, adriamycin and cisplatin. Interestingly, UV irradiation, etoposide and cisplatin all significantly induced PFKFB3 K472 acetylation (Fig. 6a). We found that cisplatin, etoposide and UV irradiation treatment increased PFKFB3 K472 acetylation in a dose- and time-dependent manner (Supplementary Fig. 8b-f). Cisplatin-induced K472 acetylation of endogenous PFKFB3 was also observed in multiple cancer cell lines (Supplementary Fig. 8g). p53 is known to be acetylated at K320 in the nucleus in response to DNA damage signal^43–45^. Intriguingly, the region near K472 of PFKFB3 displays high similarity with the sequence around K320 of p53 (Fig. 6b), consistent with the notion that both PFKFB3 and p53 are acetylated by PCAF in response to DNA damage^43,46^.

We next test whether cisplatin treatment affects PFKFB3 localization. In contrast to the nuclear localization of PFKFB3 in untreated cells, a fraction of PFKFB3 (about 10%) was found in cytoplasm upon cisplatin treatment as determined by immunofluorescent staining (supplementary Fig. 9a and 9b). Notably, this ratio is similar to that of the relative PFKFB3 acetylation induced by cisplatin treatment (12% as shown in Supplementary Fig. 7a-c). We also used another biochemical approach to demonstrate PFKFB3 translocation upon cisplatin. Digitonin is widely used for selective permeabilization of cellular and organellar membranes in animal cells^47^. We observed that treatment of HeLa cells with low concentrations of digitonin (0.015–0.03 mg/ml) could permeabilize the plasma membrane, leading to the release of cytoplasmic protein (such as glyceraldehyde 3-phosphate dehydrogenase (GAPDH) and tubulin), while keeping the nucleus intact as the nuclear protein PCNA and lamin A/C were not released (Fig. 6c). Notably, cisplatin treatment increased PFKFB3 protein that was released by digitonin, while similar treatment had no effect on the release of GAPDH or tubulin (Fig. 6c). These results further support that cisplatin treatment induces PFKFB3 cytoplasmic retention. It is worth noting that only a fraction of PFKFB3 is cytoplasmic. However, PFKFB3 is a regulatory enzyme as its product is a potent regulator of glycolysis. Moreover, PFKFB3 has a much higher ratio of kinase/phosphatase activity (710-fold), much more than the other PFKFBs. A fraction (10%) of PFKFB3 may have a big effect on glycolysis, which is supported by our data of ECAR and lactate secretion (Fig. 1a, b).

In accordance to K472 acetylation, cisplatin treatment also induced S461 phosphorylation of PFKFB3 in a dose- and time-dependent manner (Supplementary Fig. 8b, 8c). Importantly, we also observed an increase of S461 phosphorylation of endogenous PFKFB3 in multiple cell lines in response to cisplatin treatment (Fig. 6d and Supplementary Fig. 8g). Together with the finding that cisplatin treatment enhanced glycolysis in HeLa cells, and deletion of *PFKFB3* blocked this activation (Fig. 1a, b), our data indicate that genotoxic stress such as cisplatin causes acetylation and cytoplasmic accumulation of PFKFB3, leading to its activation by AMPK-mediated phosphorylation and stimulation of glycolysis. Similar to previous results, the effect of cisplatin on S461 phosphorylation was also exaggerated by either K472Q or K472R mutation (Supplementary Fig. 10), suggesting that cytoplasmic localization makes PFKFB3 more sensitive to DNA damage agent-induced phosphorylation and activation. It should be noted that DNA damage agents also directly activate AMPK^48^, which is confirmed in our experiments (as indicated by increased p-ACC in Fig. 1f). Thus full phosphorylation of PFKFB3 upon these agents may be a combined effect of both AMPK activation and acetylation-induced cytosolic accumulation of PFKFB3.

### Cisplatin induces PFKFB3 acetylation by activating PCAF/GCN5

We next investigated the mechanism of cisplatin-induced PFKFB3 acetylation. We found that cisplatin or etoposide treatment still enhanced K472 acetylation in *SIRT1* knockout cells (Fig. 6e and Supplementary Fig. 11a), while combined knockdown of *PCAF* and *GCN5* abolished cisplatin- or etoposide-induced PFKFB3 K472 acetylation (Fig. 6f and Supplementary Fig. 11b), indicating PCAF and GCN5 are responsible for this process. It has been reported that the acetylation level of GCN5/PCAF substrates (such as p53 and Histone H3) were increased after UV DNA damage^43,46,49^. Consistently, we found that the acetylation levels of p53 (K320) and Histone H3 (K14) were accumulated as PFKFB3 (K472) after cisplatin and etoposide treatment (Supplementary Fig. 11c), indicating activation of GCN5/PCAF. The activity of PCAF is known to correlates with its auto-acetylation^50,51^. We observed that acetylation level of both PCAF and GCN5 was increased in a time-dependent manner upon cisplatin treatment (Fig. 6g,h). Moreover, activation of PCAF and GCN5 upon cisplatin treatment was also demonstrated by an in vitro acetylation assay using purified PFKFB3 as the substrate (Fig. 6i). Taking together, these results suggest that cisplatin induces PFKFB3 K472 acetylation by activating the PCAF/GCN5 acetyltransferases, but not suppressing the deacetylation process.

We also explored upstream signaling machinery that mediate DNA damage signals to PFKFB3 acetylation. The phosphoinositide-3-like kinase (PIKK) family kinases, ATM, ATR and DNA-PK, are well-characterized in DNA damage response^52^. Therefore, we assessed whether these DNA damage related kinases are involved in cisplatin-induced PFKFB3 acetylation. We found that inhibition of ATM almost completely abolished cisplatin-induced K472 acetylation (Supplementary Fig. 11d), suggesting that ATM plays a major role in cisplatin-induced PFKFB3 K472 acetylation.

### PFKFB3 protects cells from cisplatin-induced apoptosis

**Fig. 7:**
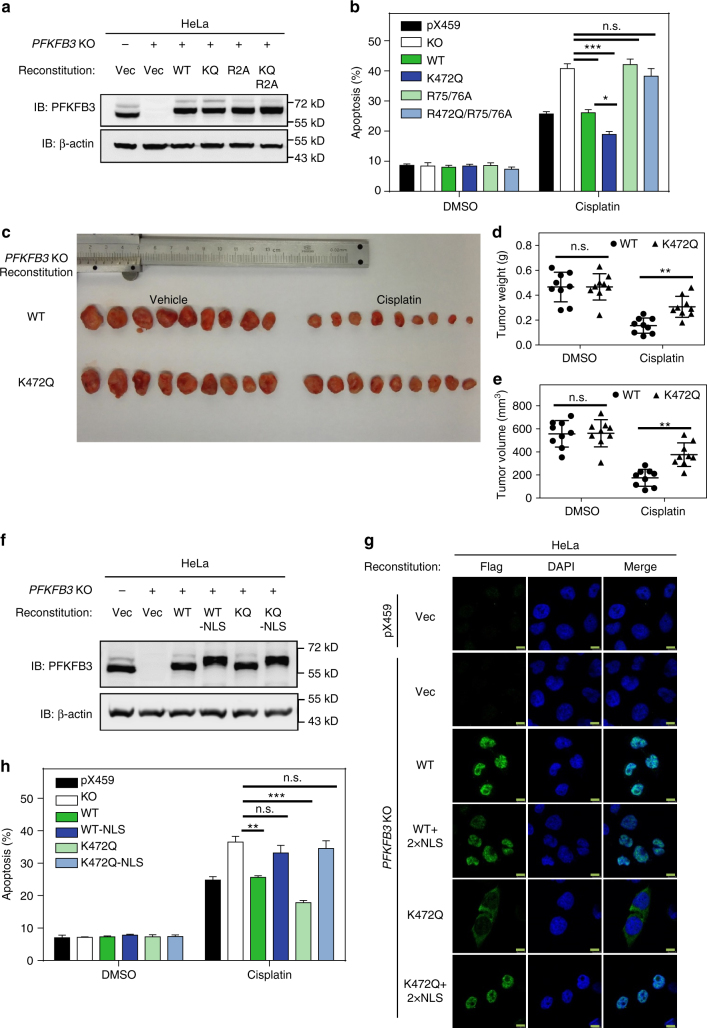
PFKFB3 protects cell from cisplatin-induced apoptosis. **a** Establishment of *PFKFB3* KO HeLa stable cells that stably express WT, K472Q, R75/76A or K472Q/R75/76A mutant of PFKFB3. Reconstitution of PFKFB3 proteins were verified by immunoblotting. **b** K472Q mutant of PFKFB3 protects cells from cisplatin-induced apoptosis in an enzyme activity-dependent manner. PFKFB3 knockout or its derived stable cells described in **a** were treated with cisplatin (10 μM) for 24 h. Cell apoptosis was assessed by flow cytometry analysis with staining of Annexin V and/or PI-positive cells. Data are presented as mean ± s.d. of five biological replicates, and statistical analyses were performed by using one-way ANOVA with Bonferroni’s post-test. *P < 0.05 and ***p < 0.001 for the indicated comparison; n.s., not significant. **c** K472Q mutant of PFKFB3 protects tumors from cisplatin-induced growth inhibition. Xenograft experiment with WT or K472Q mutant PFKFB3 cells were described in the Methods section. Xenograft tumors were collected and photographed. **d**, **e** Quantification of **d** average volume and **e** weight of xenograft tumors. Nine tumors collected from individual mice were included in each group. Data are presented as mean ± s.d. and statistical analyses were performed by using two-way ANOVA with Bonferroni’s post-test. **P < 0.01; n.s., not significant. **f** Establishment of *PFKFB3* KO HeLa stable cells that stably express Flag-tag WT, WT-NLS, K472Q, or K472Q-NLS mutant of PFKFB3. Reconstitution of PFKFB3 proteins was verified by immunoblotting with PFKFB3 antibody. **g** Additional nuclear localization sequence retain both WT and K472Q PFKFKB3 in the nuclear. Localization of reconstituted Flag-tag PFKFB3 was examined by IF staining with Flag antibody. Scale bars: 10 μm. **h** Nuclear localized PFKFB3 failed to protect cells from cisplatin-induced apoptosis. *PFKFB3* knockout or its derived stable cells described in **f** were treated with cisplatin (10 μM) for 24 h. Data are presented as mean ± s.d. of three biological replicates, and statistical analyses were performed by using one-way ANOVA with Dunnett’s post-test. **P < 0.01 and ***p < 0.001 for the indicated comparison; n.s., not significant

To provide direct evidence linking PFKFB3 K472 acetylation and cell protection, we reconstituted the PFKFB3 knockout HeLa cells with WT or K472Q mutant. The WT and mutant PFKFB3 were expressed at a level similar to endogenous PFKFB3 (Fig. 7a). We found that either WT or K472Q mutant PFKFB3 protected cells from cisplatin-induced apoptosis, with K472Q mutant being more effective than WT PFKFB3 (Fig. 7b and Supplementary Fig. 12a). Next, we examined whether the catalytic activity of PFKFB3 is required for cell protection. Arginine 75 and 76 in PFKFB3 are essential for catalytic activity and they were mutated to alanine residues to create catalytic inactive mutant R75/76A (R2A)^6^. The catalytic inactive mutant R2A or K472Q/R2A completely lost the ability to protect cells from cisplatin-induced cell apoptosis, suggesting the enzymatic activity of PFKFB3 is essential for cell protection.

Furthermore, we performed xenograft assay using *PFKFB3* knockout HeLa cells with re-expression of WT or K472Q mutant of PFKFB3. Cells expressing K472Q mutant showed significantly less sensitivity to the cytotoxicity of cisplatin than those expressing WT PFKFB3 (Fig. 7c–e). In order to further investigate the importance of cytoplasmic localization of PFKFB3, an additional NLS (sequence as “PKKKRKVD”) that is different from PFKFB3-NLS was added to both the N and C terminus of WT or mutant PFKFB3 (namely PFKFB3-NLS and K472Q-NLS, respectively). The two mutants were then stably expressed in *PFKFB3* knockout cells, and IF results confirmed that additional NLS retained both WT and K472Q PFKFB3 in the nucleus (Fig. 7g). Upon cisplatin treatment, expression of PFKFB3-NLS or K472Q-NLS was no longer able to alleviate cell apoptosis caused by *PFKFB3* knockout (Fig. 7h and Supplementary Fig. 12b). These results demonstrate that PFKFB3 cytoplasmic accumulation is important to protect cells from cisplatin treatment.

**Fig. 8:**
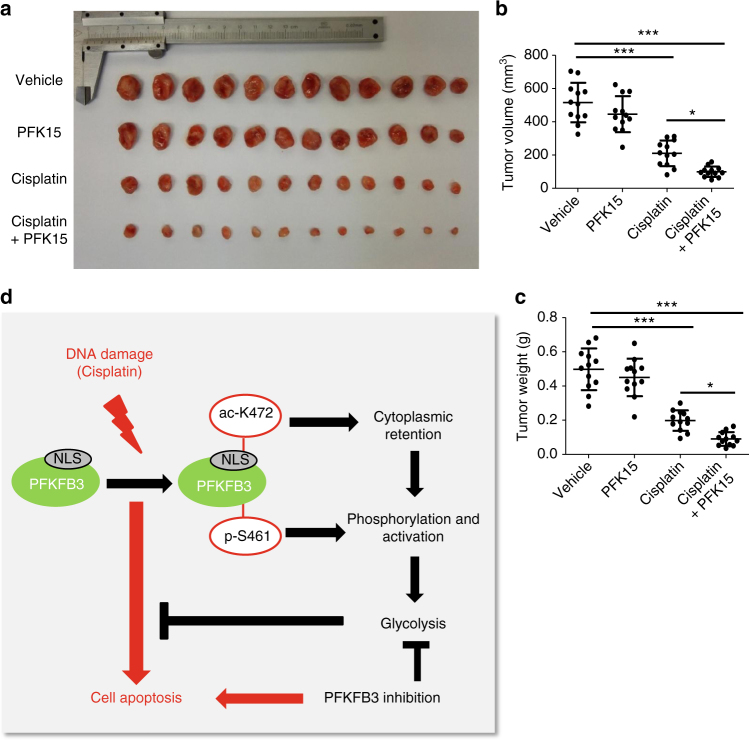
Inhibition of PFKFB3 cooperates with cisplatin to inhibit xenograft growth. **a** Combined use of cisplatin and PFK15 is more efficient in inhibiting xenograft tumor growth than single treatment. Xenograft experiment followed by cisplatin or PFK15 injection was described in the Methods section. Xenograft tumors were collected and photographed. **b**,** c** Quantification of **b** average volume and **c** weight of xenograft tumors from **a** is shown. Twelve tumors from individual mice were included in each group. Data are presented as mean ± s.d., and statistical analyses were performed by using one-way ANOVA with Bonferroni’s post-test. *P< 0.05 and ***p < 0.001 for the indicated comparison; n.s., not significant. **d** A working model that PFKFB3 K472 acetylation plays a key role in protecting cells from cisplatin-induced apoptosis

Finally, we performed xenograft tumor growth assays in nude mice subcutaneously injected with HeLa cells followed by treatment with cisplatin alone, PFK15 alone or both. The combination of cisplatin and PFKFB3 inhibition by PFK15 was more potent to inhibit tumor growth than each single treatment (Fig. 8a–c), suggesting a possible benefit of combining PFKFB3 inhibitors with cisplatin therapy (Fig. 8d).

## Discussion

Phosphorylation-mediated activation of PFKFB3 in response to multiple external stresses has been extensively studied^13,14,19,53,54^. To the best of our knowledge, this study represents the first report of the regulation of PFKFB3 by acetylation. Importantly, we have determined the physiological signal, the function consequence and the mechanism of PFKFB3 acetylation. We found that K472 acetylation of PFKFB3 is stimulated by cisplatin-induced nuclear DNA damage, which impairs PFKFB3 translocation to the nucleus and accumulates PFKFB3 in the cytoplasm where it is phosphorylated and activated. Higher PFKFB3 activity increases F2,6BP and stimulates glycolysis, leading to increased cell protection^13^. We elucidated the mechanism by which K472 acetylation regulates nuclear and cytoplasmic distribution of PFKFB3. Although localization of endogenous PFKFB3 was not determined because of lack of a suitable antibody, PFKFB3 ectopically expressed at a level similar to the endogenous one could represent the regulation of PFKFB3 localization (Fig. 7g). We discovered that importin 5α is the major importin for PFKFB3 nuclear transportation and acetylation of K472 within the NLS abolishes the interaction of the PFKFB3-NLS peptide with importin 5α. We have further identified both the deacetylases, SIRT1, and the acetyltransferase, PCAF/GCN5, that play the key role in controlling K472 acetylation. PCAF was previously found to acetylate p53 in response to DNA damage at K320^46^, which is surrounded by a sequence that is very similar to the sequence surrounding the K472 of PFKFB3 (Fig. 6b). It remains to be determined how DNA damage stimulates the interaction of PCAF with either p53 or PFKFB3.

Reprogramming of energy metabolism is a hallmark of cancer. Tumor cells may manipulate metabolic regulation to gain growth advantage or to evade apoptosis and cell death^55^. Enhanced cytoplasmic glycolysis has been reported as a cell protection mechanism following mitochondrial damage, presumably to increase ATP production and compensate impaired ATP production in mitochondrial^13^. How tumor cells regulate glycolysis to evade apoptosis induced by nuclear DNA damage is still unknown. Our work suggests that nuclear localized PFKFB3 may serve as an important node in sensing nuclear stress and signaling cytoplasmic glycolytic control that may have been exploited by tumor cells. Inhibition of PFKFB3 with chemical inhibitors or genetic silence reduces glycolysis rate and suppresses tumor cell proliferation^7–9,24^. A small-molecule antagonist of PFKFB3, 3PO (3-(3-pyridinyl)-1-(4-pyridinyl)-2-propen-1-one), can promote breast cancer cell death during microtubule poison-induced mitotic arrest^13^. We found that inhibition PFKFB3 by PFK15, which is about 100-fold more potent than 3PO toward PFKFB3^24^, cooperates with cisplatin to promote cancer cell apoptosis and significantly potentiates the inhibitory effect of cisplatin on tumor growth, providing further evidence that PFKFB3 inhibitor, such as PFK15, merits to be exploited in cancer treatment.

Cisplatin is a widely used first-line chemotherapy in treating multiple human malignancies by inducing DNA damage-triggered cell-cycle arrest and apoptosis^22,23^. However, the requirement of cisplatin-sensitization strategies constitute a goal with important clinical implications due to the existence of drug resistance^23^. We found here that upregulation of glycolysis is an important way for cancer cells to evade cisplatin-induced apoptosis, which is partially supported by previous studies that inhibition of glycolysis promotes cisplatin-induced cell apoptosis^56,57^. We uncovered a novel mechanism by which cisplatin stimulates glycolysis: cisplatin induces PFKFB3 acetylation and accumulation in the cytoplasm where it potently promotes glycolytic flux. Importantly, inhibition of PFKFB3 by small-molecule PFK15 effectively enhances cisplatin-induced apoptosis of cancer cells. PFKFB3 is broadly expressed in multiple tissues and many different types of cancers^58^, suggesting it may be a potential drug target for multiple cancers. In fact, a very recent study reported that inhibition of PFKFB3 improves cisplatin chemotherapy on melanoma tumor growth and metastasis in a xenograft model, possibly through promoting tumor vessel normalization and drug delivery^12^. Our findings reinforces the concept that combined therapy of cisplatin with radiotherapy or other chemotherapeutic agents such as paclitaxel, gemcitabine and vinorelbine achieve better clinical effect^59,60^ and identifies a potential new agent that could potentiate the antitumor effect of cisplatin. In addition, our finding that etoposide and UV also stimulate K472 acetylation of PFKFB3 suggest a potential broad applicability of PFK15 to enhance the efficacy of additional chemotherapeutic agents for the treatment of different types of tumor.

## Methods

### Antibodies

Antibodies against pan-acetylated lysine (9441, 1:1000), phospho-(Ser/Thr) AMPK Substrate (5759, 1:1000), ACC (3676, 1:2000), phospho-Acetyl-CoA Carboxylase (Ser79, 11818, 1:2000), AMPKα (2532, 1:2000), phospho-AMPKα (Thr172, 2535, 1:1000), Histone H3 (4499, 1:5000), acetyl-Histone H3 (Lys14, 7627, 1:1000), phospho-Histone H2A.X (Ser139, 9718, 1:2000), Tubulin (2144, 1:1000), PCNA (2586, 1:1000) and GCN5 (3305, 1:1000) were purchased from Cell Signaling Technology. Antibody against acetyl-p53 (Lys320, 06–1283, 1:1000) was purchased from Merck Millipore. Antibodies against Flag (4110, 1:3000), Lamin A+C (A01455, 1:1000) and β-actin (A00702, 1:5000) were purchased from Genescript. Antibody against HA (sc-7392, 1:1000),GAPDH (sc-32233, 1:1000) and p53 (sc-126, 1:2000) was purchased from Santa Cruz. Antibody against Myc (1208, 1:2000) was purchased from Hua An Biotechnology. Antibody against PFKFB3 (181861, 1:3000), importin α5 (55387, 1:1000) and phospho-ATM (S1981, 81292, 1:1000) was purchased from Abcam. Antibody against ATM (70103, 1:500) was purchased from GeneTex. Antibodies against SIRT1 (1104, 1:1000) and PCAF (1247, 1:1000) were purchased from Epitomics.

To generate a site-specific antibody to detect the acetylated K472 of PFKFB3 (α-acK472, 1:500), synthesized peptide SPEPTK(Ac)KPRINS (Shanghai Genomic Inc.) was coupled to KLH as antigen to immunize rabbit. Anti-serum was collected after four doses of immunization.

### Plasmids

Full-length complementary DNA (cDNA) of PFKFB3 was cloned into the pRK7-N-FLAG or pQCXIH vector using standard protocols. KPNA1, KPNA2, KPNA3, KPNA4, KPNA5 and KPNA6 cDNAs were kind gifts from Dr. Jiahuai Han’s Lab (Xiamen University, Xiamen, China) and sub-cloned into pcDNA3-HA for expression. Point mutations of the indicated constructions were generated by standard site-directed mutagenesis kit (AccuPrime™ *Pfx* DNA Polymerase, ThermoFisher). All constructions were confirmed by DNA sequencing before further applications.

### Chemicals

Nicotinamide and digitonin were purchased from Sigma-Aldrich. Tricostatin A was purchased from Cell Signaling Technology. Cisplatin, PFK15, etoposide and adriamycin were purchased from Selleck. Lambda phosphatase was purchased from New England Biolabs. Biotinylated PFKFB3 peptides containing either acetylated or non-acetylated K472 residue was purchased from GL Biochem Ltd.

### UV irradiation

HEK293T cells were cultured to approximately 70–80% confluence in 35 mm diameter dishes and were irradiated with 10 J/m^2^ UVC delivered via a HL-2000 HybriLinker with a 254 nm wavelength (Upvon), followed by the indicated recovery time before harvest.

### Cell culture and transfection

HEK293T, U2OS, A549, HCT116 and HeLa cells were purchased from the American Type Culture Collection (ATCC). HEK293T and HeLa cells were cultured in Dulbecco’s modified Eagle’s medium (GIBCO). U2OS cells were cultured in RPMI-1640 medium (Invitrogen). HCT116 cells were cultured in McCoy’s 5a Medium (Sigma). A549 cells were cultured in DMEM/F12 Medium (GIBCO). All of the above cells were supplemented with 10% fetal bovine serum (Gibco) and 50 μg/ml penicillin/streptomycin. Plasmid transfection was carried out by using polyethylenimine or lipofectamine 2000 (Invitrogen). Five cell lines were authenticated by short tandem repeat profiling by ATCC: HEK293T, U2OS, A549, HCT116 and HeLa. HEK293T, U2OS, A549, HCT116 and HeLa were tested for mycoplasma contamination and were negative.

### Immunoprecipitation and western blotting

Cells were lysed in ice-cold NP-40 buffer containing 50 mM Tris-HCl (pH 7.4), 150 mM NaCl, 0.5% NP-40, 1 μg/ml Aprotinin, 1 μg/ml Leupeptin, 1 μg/ml Pepstatin, 1 mM Na_3_VO_4_, 50 mM NaF and 1 mM phenylmethylsulfonyl fluoride. Cell lysate was centrifuged at 12,000×*g* for 15 min at 4 °C. The supernatant was incubated with Flag beads (Sigma) for 3 h at 4 °C, or with indicated antibody for 2 h followed by incubation with Protein-A beads (Upstate) for another 2 h at 4 °C. After washing three times with ice-cold NP-40 buffer, the proteins were denatured by SDS loading buffer containing dithiothreitol.

For acetylation western blotting, 50 mM Tris (pH 7.5) with 10% (v/v) Tween-20 and 1% peptone (AMRESCO) was used for blocking, and 50 mM Tris (pH 7.4) with 0.1% peptone was used to prepare primary and secondary antibodies.

Immunoblotting intensity was quantified using Image Quant TL software (GE Healthcare). Unprocessed blot images are shown in Supplementary Fig. 13.

### Identification of PFKFB3-acetylated sites

Flag-PFKFB3 proteins were expressed in HEK293T cells and immunopurified from cells, resolved on 10% SDS–polyacrylamide gel electrophoresis, and stained by Coomassie blue and sliced. The dye of gel slice was removed by soaking with 50 mM NH_4_HCO_3_ and 50% acetonitrile, followed by water wash twice and removing water by acetonitrile. The gel was dried and digested in 100 ml 50 mM NH_4_HCO_3_ with trypsin (trypsin/protein at 1:30) at 37 °C overnight. The trypsin-treated peptides were extracted by a volume containing 50% acetonitrile and 0.1% trifluoroacetic acid and then followed by vacuum dry. The peptides were resuspended with solvent A (A: water with 0.1% formic acid), separated by nano-liquid chromatography and analyzed by online electrospray tandem mass spectrometry. The experiments were performed on a Nano Aquity UPLC system (Waters Corporation, Milford, MA) connected to a quadrupole-Orbitrap mass spectrometer (Q-Exactive) (Thermo Fisher Scientific, Bremen, Germany) equipped with an online nano-electrospray ion source.

### Generation of knockout cells using CRISPR/Cas9 genome editing

Two independent clones with *PFKFB3* or *SIRT1* gene deletion were used for experiments. The detail protocol has been described elsewhere^61^. Gene deletion was verified by both Sanger sequencing of genomic DNA and western blotting for cell lysate. The guide sequences targeting the human *PFKFB3* and *SIRT1* gene are shown below:

*PFKFB3* #1: 5ʹ- AGCTGACTCGCTACCTCAAC-3ʹ

*PFKFB3* #2: 5ʹ- GCCCACCATGACGATGACGG-3ʹ

*SIRT1* #1: 5ʹ- GACACGCTGGAACAGGTTGC-3ʹ

*SIRT1* #2: 5ʹ- AGTGTCATATCATCCAACTC-3ʹ.

### Retroviral infection

To generate U2OS cells stably expressing green fluorescent protein (GFP)-fused WT, K472Q or K472R mutants of PFKFB3, retroviruses carrying pBABE-GFP vector or pBABE-GFP-WT, K472R or K472Q of PFKFB3 were produced in HEK293T cells using VSVG and GAG as packaging plasmids. Retroviral supernatant was harvested 36 h after initial plasmid transfection and mixed with polybrene (8 μg /ml) to increase the infection efficiency. Stable cell pools were selected with 1 μg/ml puromycin (Amresco) for 3 days.

To generate HeLa cells stably expressing WT, K472R, K472Q, R75/76A or R75/76A/K472Q of PFKFB3, retroviruses carrying pQCXIH empty vector or pQCXIH WT, K472R, K472Q, R75/76A or R75/76A/K472Q of PFKFB3 were produced in HEK293T cells using VSVG and GAG as packaging plasmids. Virus supernatant was filtered through 0.45 µm filter and infected HeLa *PFKFB3* knockout cells in the presence of 8 µg/ml polybrene. Stable cell pools were selected with 50 μg/ml hygromycin B (Amresco) for 5 days.

### RNA interference

The siRNAs targeting *PCAF and GCN5* were from Genepharma and were delivered into cells using RNAiMAX (Invitrogen) according to the manufacturer’s instruction. The sequences of siRNAs used in this study were shown below:

si*PCAF* #1: GCATCCAAACAGTTATCAA

si*PCAF* #2: CCGTATGTTCCCATCTCAA

si*GCN5* #1: GCATGCCTAAGGAGTATAT

si*GCN5* #2: TCTTCTACTTCAAGCTCAA.

### Measurement of intracellular ATP levels

Cellular ATP levels were determined by using a commercial ATP Colorimetric Assay Kit (Sigma-Aldrich). Briefly, 1 × 10^6^ cells were lysed with ATP assay buffer and deproteinized by using a 10 kDa MWCO spin filter. ATP assay buffer containing ATP probe and converter was added to the sample at room temperature for 30 min, and then measured the absorbance at 570 nm (A_570_) in a microplate reader. All samples and standards were run in triplicate.

### Immunofluorescence staining

Cells seeded in 12-well plates were treated as indicated in specific experiments. After treatment, they were fixed immediately with 4% paraformaldehyde for 30 min and then permeabilized with 0.2% Triton X-100 in phosphate-buffered saline (PBS) for 10 min. After blocking in 5% bovine serum albumin for 60 min, cells were incubated with primary antibodies overnight at 4 °C. Cells were washed with PBS 3 times for 5 min, and Alexa Fluor 488-conjugated secondary antibodies were added for 1 h at room temperature. 4',6-diamidino-2-phenylindol (DAPI) was used to stain cell nuclei. Images were captured on an Olympus IX81 Inverted Research Microscope or a Leica TCS SP8 Microscope. Immunofluorescence signal was analyzed and quantified using ImageJ software.

### Cell plasma membrane permeabilization

Cells were suspended with PBS in Eppendorf tubes, followed by incubation with low concentrations of digitonin (0.015–0.03 mg/ml) at 37 °C for 3 min. Then, Eppendorf tubes were centrifuged at 2000×*g* for 2 min at 4 °C to separate the supernatant and precipitate.

### Flow cytometry

Cell apoptosis was assayed by using a commercial FITC Annexin V Apoptosis Detection Kit (BD Pharmingen). Cells were washed twice with cold PBS and then resuspended at a concentration of 1 × 10^6^ cells/ml. Then, 5 μl of FITC Annexin V and 5 μl propidium iodode (PI) were added into 100 μl of cell suspension followed by incubation at 25 °C for 15 min in the dark. The percentages of Annexin V and/or PI-positive cells were determined by flow cytometer (BD Accuri^TM^ C6).

### Extracelluar acidification rate

ECAR was assayed by using a Seahorse XF96 instrument (Seahorse Biosciences) according to the manufacturer’s instructions. Cells were counted and seeded into the Seahorse 96-well plate at a density of 8000 cell/well, followed by culturing for 12 h. After that, 10 mM glucose, 2 μM oligomycin and 2-deoxy-d-glucose were added into different ports of the Seahorse cartridge.

### Phosphofructokinase activity assay

The activity of endogenous phosphofructokinase was measured using Phosphofructokinase Activity Colorimetric Assay Kit (Bio Vision, catalog K776). In brief, 2 × 10^6^ cells were lysed in 100 μl of PFK Assay Buffer on ice. Small molecules in samples were removed by 10 kD spin column (Abcam, catalog ab93349) and 5 μl of lysate was used per assay. Absorbance was measured at 450 nm for two time points. The activity of PFK-1 was calculated by dividing the amount of generated NADH by the reaction time and was normalized against protein concentration.

### Lactate production assay

Lactate was quantified using Bio Vision Lactate Assay Kit (Bio Vision, catalog K607). In brief, 0.5 μl of culture medium collected from treated cells was add to Lactate Assay Buffer. The reaction was incubated for 30 min at room temperature, protected from light. Absorbance was measured at 570 nm a microplate reader. Lactate production was indexed to the number of cells.

### In vitro acetylation assay

In vitro PFKFB3 acetylation assays were performed using His-tag PFKFB3 proteins purified from *Escherichia coli* BL21, and Flag-tag PCAF/GCN5 acetyltransferases were purified from HEK293T cells treated with or without cisplatin. His-tag PFKFB3 proteins were incubated with PCAF/GCN5 in 30 μl reaction buffer (50 mM Tris-HCl (pH 8.0), 50 mM NaCl, 4 mM MgCl_2_, 0.1 mM EDTA, 1 mM DTT and 10% glycerol) in the presence or absence of the acetyl-coenzyme A (CoA) (2 mM) at 37 °C for 30 min.

### Xenograft

Female nude mice (5 weeks old) were purchased from SLAC and maintained and treated under specific pathogen-free conditions. All animal-related procedures were performed under the Division of Laboratory Animal Medicine regulations of Fudan University. For Fig. 7c, the mice were injected subcutaneously with 6 × 10^6^ *PFKFB3* knockout HeLa cells re-expressed WT or K472Q mutant of PFKFB3. When tumors reached a size of 5–6 mm in diameter, they were randomly divided into control group and cisplatin treatment group (9 mice per group). Cisplatin was administered by intraperitoneal (i.p.) injection at 5 mg per kg per week for 2 weeks. For Fig. 8a, the mice were injected subcutaneously with 6 × 10^6^ HeLa cells. When tumors reached a size of 5–6 mm in diameter, they were randomly divided into a control group and treatment groups including PFK15 alone, cisplatin alone and PFK15 in combination with cisplatin for 15 days (12 mice per group). The sample size was based on our extensive experience in analyzing mouse xenografts. No animals were excluded from the analysis. The investigators were not blinded to group allocation. PFK15 was administered by i.p. injection at 25 mg per kg per 3 days. Cisplatin was administered by i.p. injection at 5 mg per kg per week. Tumor volume=(π/6) (*W*^2^) (*L*), where *W* represents width, and *L* represents length.

### Statistical analysis

Results were analyzed and graphed using Prism 7.0 software (Graphpad Software). All data shown represent the results obtained from at least three biological or technical replicates as indicated with standard deviation of the mean (mean ± s.d.). The sample sizes were chosen to allow for statistical significance testing assuming a major effect and a small variation. The variance was similar between the compared groups. Statistical analyses were performed using one-way or two-way analysis of variance (ANOVA) with Bonferroni’s test or Dunnett’s test as indicated in corresponding figure legends. *P* < 0.05 was considered statistically significant.

### Data availability

The data that support the findings of this study are available in this manuscript and its supplementary information or from the corresponding author on request.

## Electronic supplementary material

Supplementary Information
