# Supplementary material for: Acetylation accumulates PFKFB3 in cytoplasm to promote glycolysis and protects cells from cisplatin-induced apoptosis
Source: Nat Commun. 2018 Feb 6;9:508. doi: 10.1038/s41467-018-02950-5 (PMC5802808; doi:10.1038/s41467-018-02950-5)

Supplementary Information

Title: Acetylation accumulates PFKFB3 in cytoplasm to promote glycolysis and protects cells from cisplatin-induced apoptosis

Li et al.

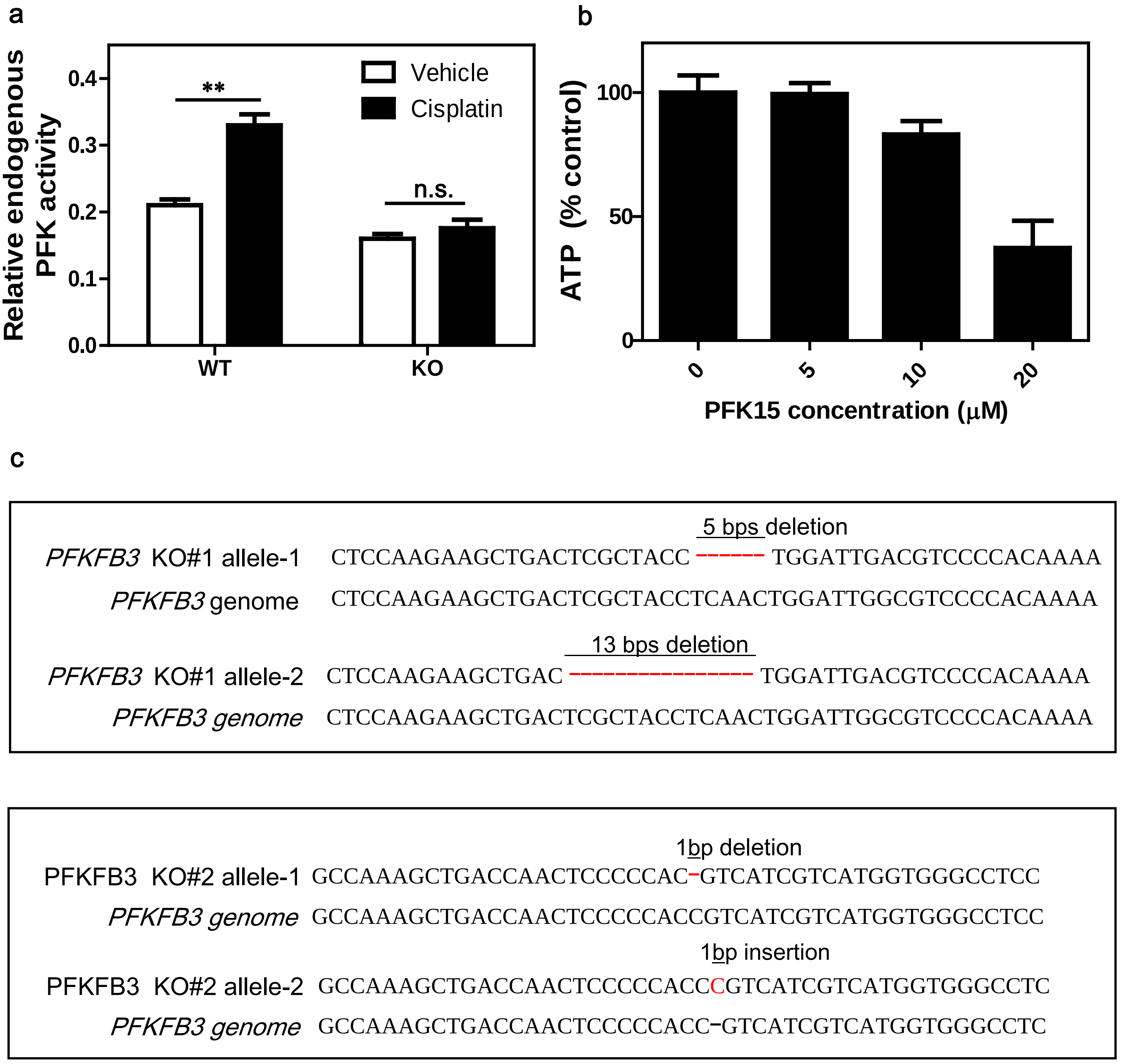

Supplementary Fig. 1 Inhibition of PFKFB3 cooperates with cisplatin to promote cancer cell apoptosis
(a) Cisplatin treatment enhanced PFK activity. Endogenous PFK activity were measured in WT or *PFKFB3* KO HeLa cells treated with or without cisplatin (10 μM) for 24 h. Data are presented as mean ± s.d. of three biological replicates, and statistical analyses were performed by using two-way ANOVA with Bonferroni's post-test. ** denotes $p < 0.01$, n.s., not significant.
(b) PFK15 treatment reduces intracellular ATP level. HeLa cells were exposed to PFK15 at the indicated concentrations for 24 h. Intracellular ATP levels were measured as described in the Methods. Data are presented as mean ± s.d. of three biological replicates.
(c) Verification of HeLa cell lines with *PFKFB3* deletion. Alignment of genomic sequence of *PFKFB3* from *PFKFB3* KO cells and WT cells is shown. Both clones are heterozygous. Clone PFKFB3-KO#1: the two alleles contain 5 or 13 nucleotides deletion, respectively. Clone PFKFB3-KO#2: one allele contains one nucleotide deletion, while the other allele contains one nucleotide insertion.

a

Acetylation sites Identified by MS/MS spectrum	
K12	VQKacWVPVDHRPSLPR
K284	FASALSKacFVEEQNLK
K302	VWTSQLKacSTIQTAEALR
K451	KacGPNPLMR
K472	RNSVTPLASPEPTKacKPR
K473	NSVTPLASPEPTKKacPR

c

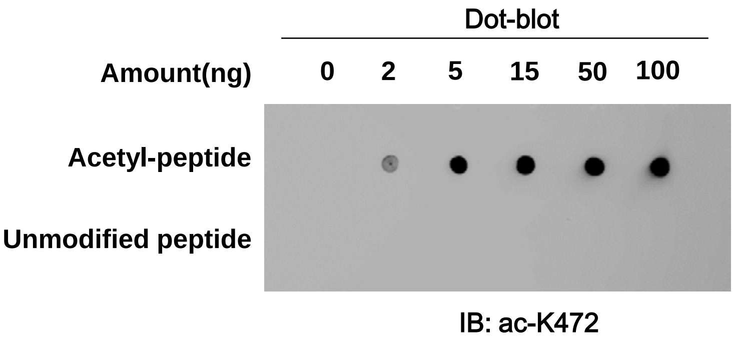

b

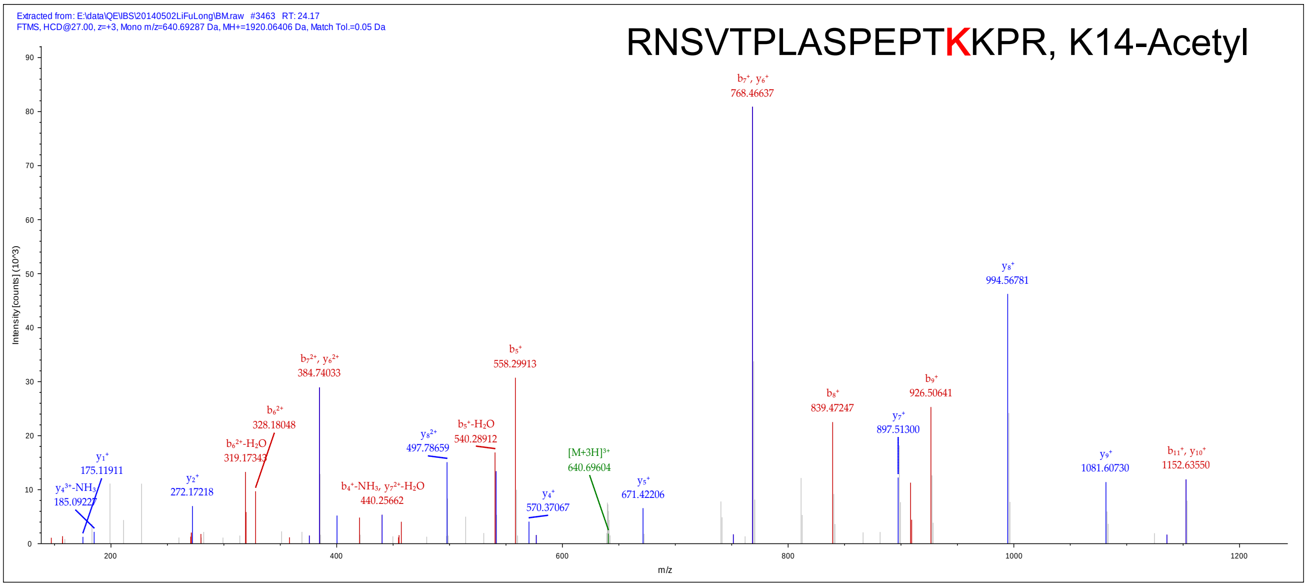

Supplementary Fig. 2 PFKFB3 is acetylated at K472

(a) Identification of acetylated PFKFB3 peptides by tandem liquid chromatography–tandem mass spectrometry (LC/LC-MS/MS).

(b) Tandem mass spectrum for the acetylated PFKFB3 K472-containing peptide is shown.

(c) Characterization of anti-acetyl-PFKFB3 (K472) (α-acK472) antibody. Specificity of antibody against acetylated K472 residue of PFKFB3 was determined by dot blot assay. Nitrocellulose membrane was spotted with different amounts of acetyl-K472 peptide or unmodified peptide and immunoblotted with anti-acetyl-PFKFB3 (K472) antibody.

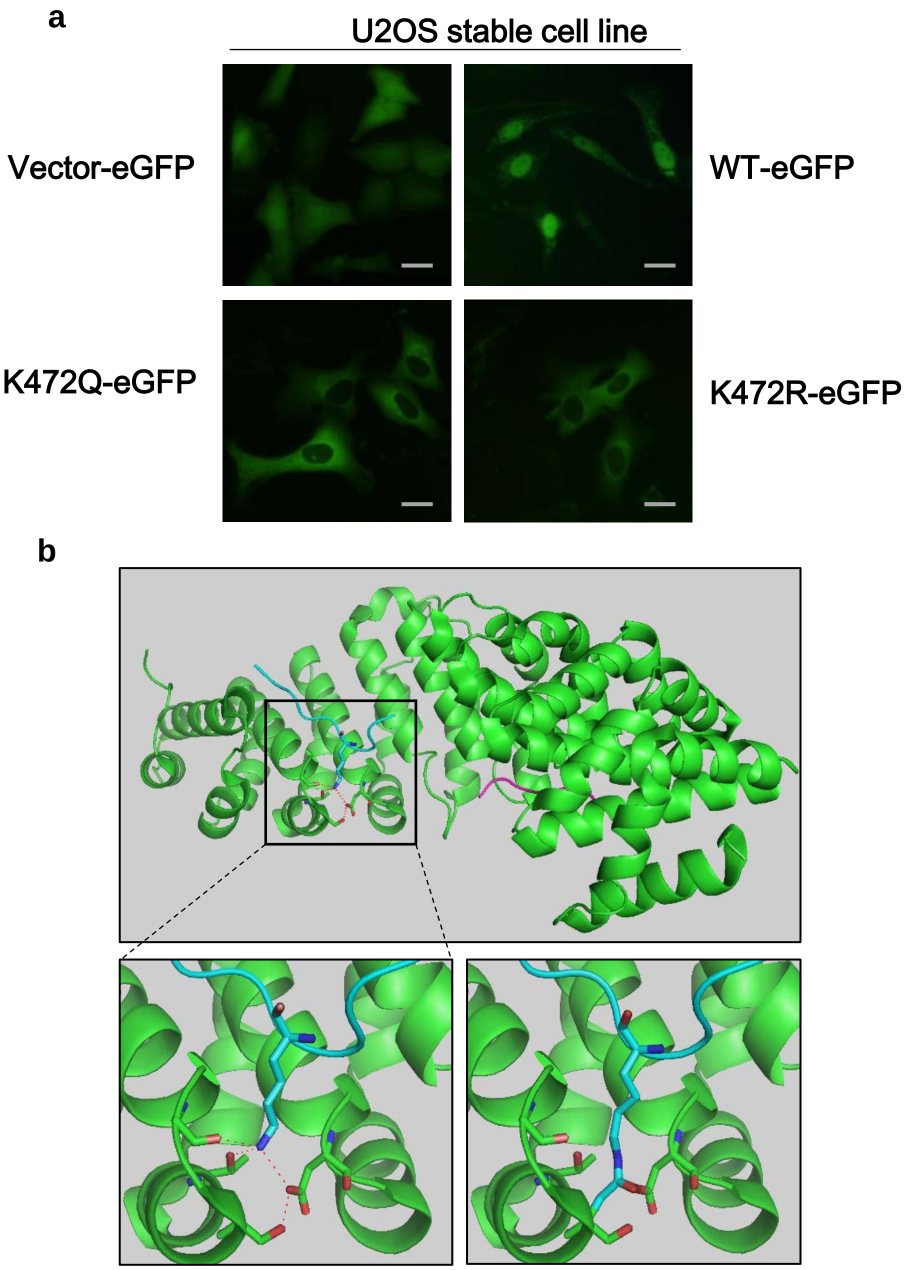

Supplementary Fig. 3 Acetylation Mimetic Mutant of PFKFB3 disrupts its NLS motif

(a) Subcellular localization of GFP fused PFKFB3 wild-type (WT), K472Q or K472R mutants stably expressed in U2OS cells was examined by detecting GFP fluorescence. Scale bars: 20 μm.

(b) Molecular modeling shows acetylation of the first lysine within NLS disrupts its recognition by the importin α subunit. Upper panel, cartoon representation of the crystal structural of mouse importin α1 bound to the classic NLS peptide (PDB ID: 3L3Q) made by the tool from Pymol (www.pymol.org). Lower left, a closer view showing the structure of peptide containing a classic NLS (KKRR) bound to importin α subunit. Lower right, the structure that is mimetic of first lysine acetylation of NLS shows the disruption of its interaction with importin α subunit. The importin α protein is colored in green and the classic NLS peptide is in blue. The red dashed lines represent critical electrostatic interactions between the lysine side chain and the oxygen of importin α subunit.

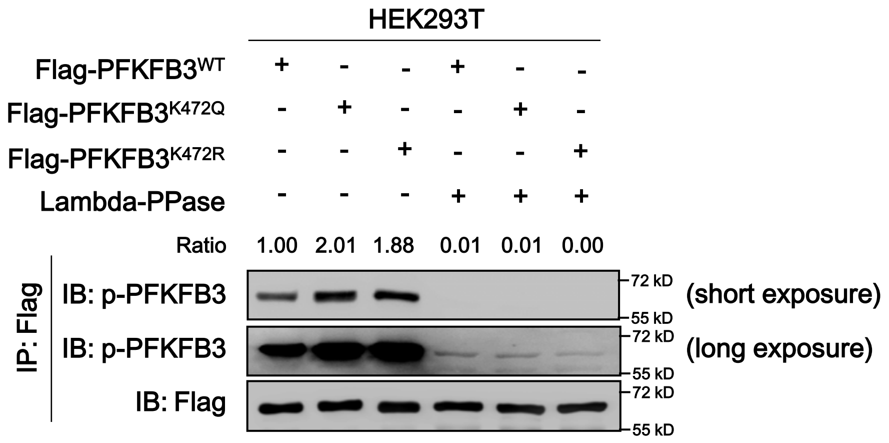

Supplementary Fig. 4 Cytoplasmic PFKFB3 potentiates S461 phosphorylation

Phosphorylation of PFKFB3 was abolished by Lambda phosphatase treatment. Flag-tagged WT, K472Q or K472R mutant of PFKFB3 was transiently expressed in HEK293T cells. PFKFB3 was immunoprecipitated with Flag beads and incubated with or without Lambda phosphatase for 30 min at 30 ^{o}C. Phosphorylation of PFKFB3 was detected with a phospho-AMPK substrate motif antibody. Relative PFKFB3 phosphorylation was normalized by Flag protein.

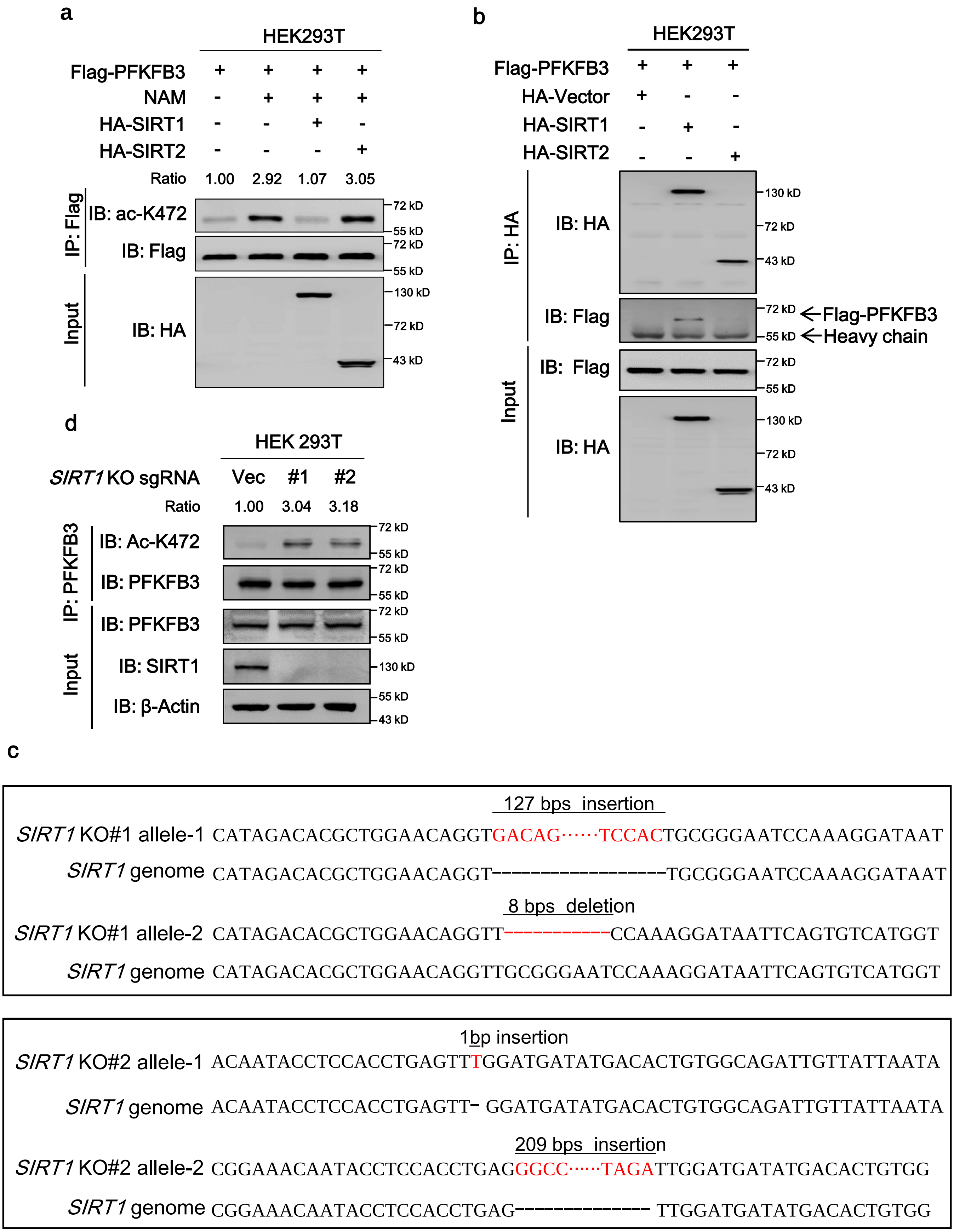

Supplementary Fig. 5 SIRT1 is the potential deacetylase for PFKFB3

(a) SIRT1 overexpression decreases K472 acetylation of PFKFB3. Flag-tagged PFKFB3 was co-expressed with either HA-tagged SIRT1 or SIRT2 in HEK293T cells. Cells were treated with low dose of NAM (2.5 mM) for 6 h. Flag-PFKFB3 was immunoprecipitated and the K472 acetylation was determined with α-AcK472 antibody. Relative PFKFB3 K472 acetylation was normalized by Flag protein.

(legend continued on next page)

(b) PFKFB3 interacts with SIRT1, but not SIRT2. Flag-tagged PFKFB3 was co-expressed with empty vector or individual HA-tagged SIRTs as indicated in HEK293T cells. SIRTs were immunoprecipitated with HA beads, followed by immunoblotting with Flag antibody to detect PFKFB3.

(c) Verification of HEK293T cell lines with *SIRT1* deletion. Alignment of genomic sequence of *PFKFB3* from *PFKFB3* KO cells and WT cells is shown. Both clones are heterozygous. Clone SIRT1-KO#1: one allele contains 127 nucleotides insertion, while the other allele contains 8 nucleotides deletion. Clone SIRT1-KO#2: the two alleles contain 1 or 209 nucleotides insertion, respectively.

(d) *SIRT1* deletion increases K472 acetylation of endogenous PFKFB3. Endogenous PFKFB3 protein was immunoprecipitated from HeLa wild-type and two *SIRT1* knockout cell lines. Immunoblotting was performed with the indicated antibodies. Relative PFKFB3 K472 acetylation was normalized by PFKFB3 protein.

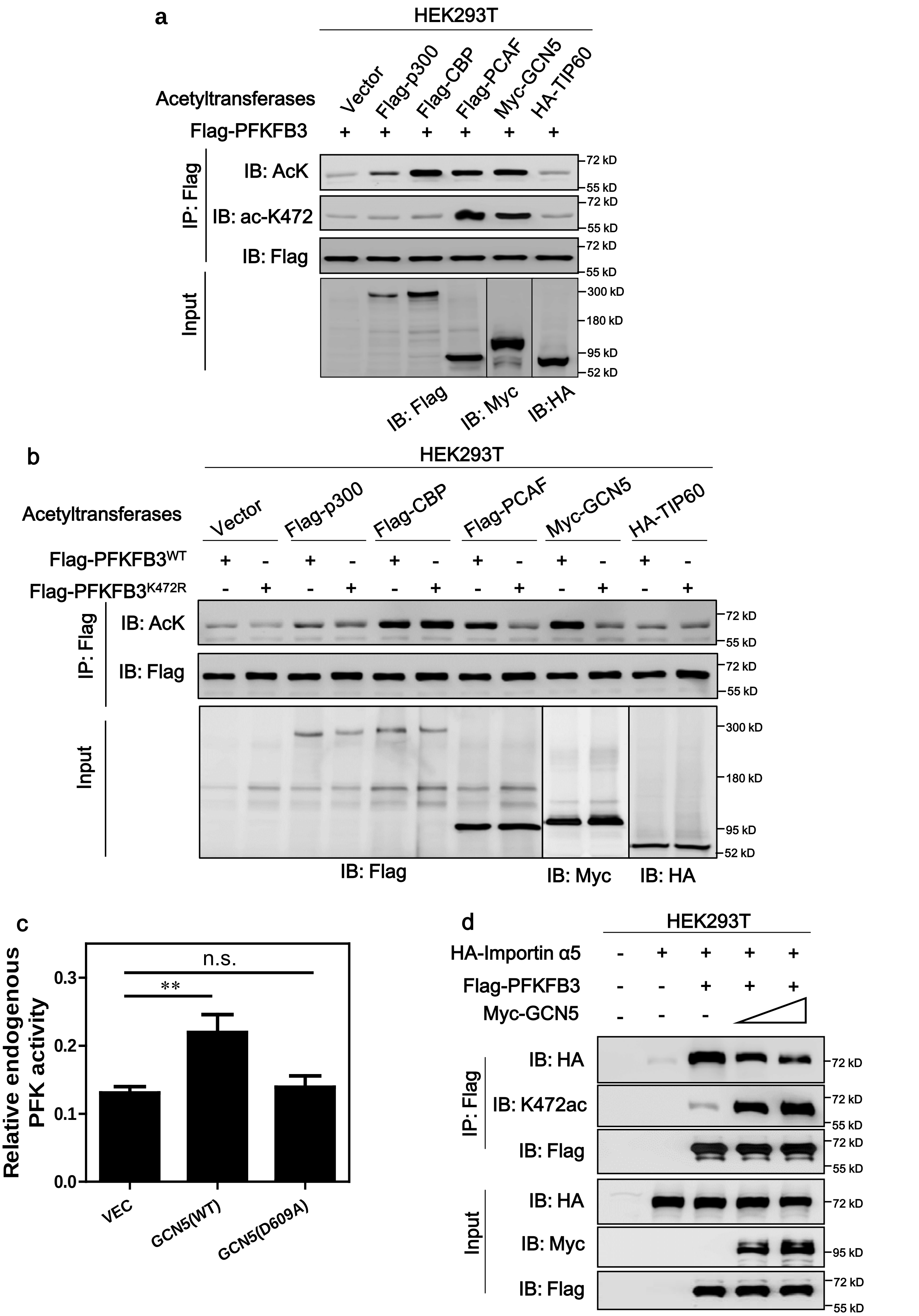

Supplementary Fig. 6 PCAF/GCN5 are the major acetyltransferases for K472 of PFKFB3

(a) Ectopic expression of PCAF and GCN5 increases K472 acetylation of PFKFB3. Flag-tagged PFKFB3 was co-expressed with different acetyltransferases indicated in HEK293T cells and purified with Flag beads. K472 acetylation of Flag-PFKFB3 was determined with α-acK472 antibody.

(legend continued on next page)

(b) K472R mutant blocks PCAF and GCN5 induced the acetylation of PFKFB3. Flag-tagged wild-type (WT) or K472R mutant of PFKFB3 was co-expressed with different acetyltransferases indicated and immunoprecipitated by Flag beads. Acetylation of PFKFB3 was examined with the α-AcK antibody.

(c) Ectopic expression of GCN5 increases endogenous PFK activity. Endogenous PFK activity were assayed in HEK293T cells transfected with pCDNA3-Myc empty vector, GCN5 wildtype or acetyltransferase activity-dead mutant of GCN5 (D609A). Data are presented as mean ± s.d. of three biological replicates, and statistical analyses were performed by using one-way ANOVA with Dunnett's post-test. *** denotes $p < 0.001$ for the indicated comparison; n.s., not significant.

(d) Co-expression of GCN5 disrupts PFKFB3 binding with importin α5. Flag-PFKFB3 and HA-importin α5 were co-transfected with empty vector or different dose of Myc-tag GCN5 construct in HEK293T cells. After 36 h, cells were lysed and immunoprecipitated with Flag beads. Immunoblotting was performed with the indicated antibodies.

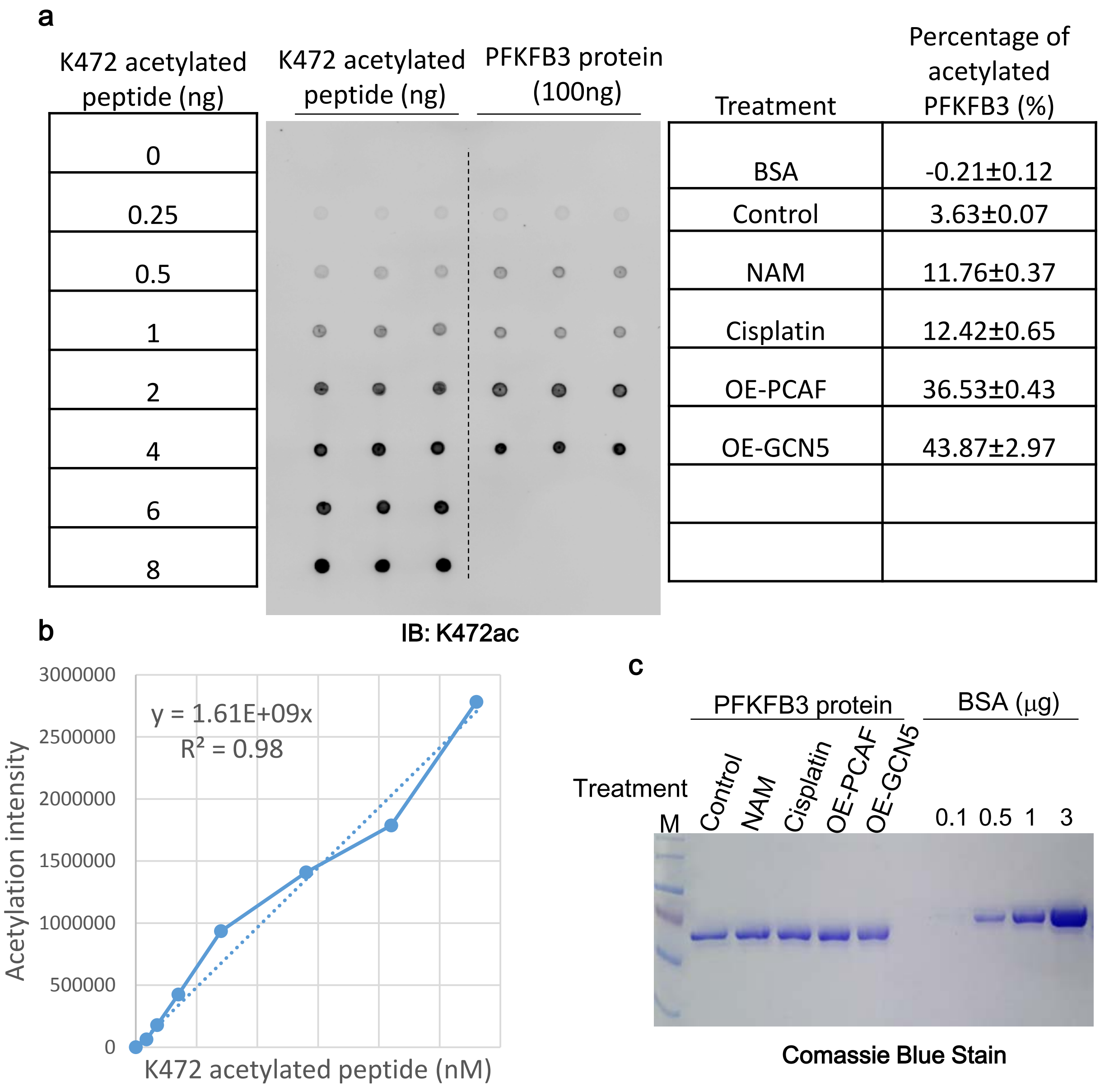

Supplementary Fig. 7 Quantification of acetylation level of PFKFB3 K472 residue upon different treatment

(a) Quantification of acetylation level of PFKFB3 K472 residue upon different treatments. Flag-tagged full length PFKFB3 protein was expressed in HEK293T cells. Cells were treated with NAM (5 μM, 6h), cisplatin (50 μM, 24 h) before harvesting, or co-transfected with PCAF or GCN5 as described in the right box. Flag-PFKFB3 was immunoprecipitated with Flag beads, eluted by 3×Flag peptide and quantified by Coomassie blue staining as described in (c). Triplicates of 100 ng of purified Flag-PFKFB3 protein were immobilized on the nitrocellulose membrane and blotted with an α-acK472 antibody. To generate a standard curve, different amounts of K472-acetylated peptide (dissolved in the elution buffer plus 100ng/μl BSA) were immobilized to the left side of the same filter. The amount of peptide in each row was described in left box. The percentage of acetylated PFKFB3 was calculated as following: The acetylation intensity of Flag-PFKFB3 was quantified by Image Quant TL software (GE Healthcare). The percentage of acetylated Flag-PFKFB3 was calculated using the standard curve derived from K472-acetylated peptide in (b). Data are presented as mean ± s.d. of three technical replicates. OE: overexpression

(b) Standard curve of K472 acetylated peptide showing the correlation between peptide amount and acetylation signal intensity in (a).

(c) Flag-PFKFB3 protein eluted in (a) was resolved by SDS-PAGE together with different amount of BSA (0.1-3 μg). Gels were stained with Coomassie blue and Flag-PFKFB3 was quantified according to standard amount of BSA.

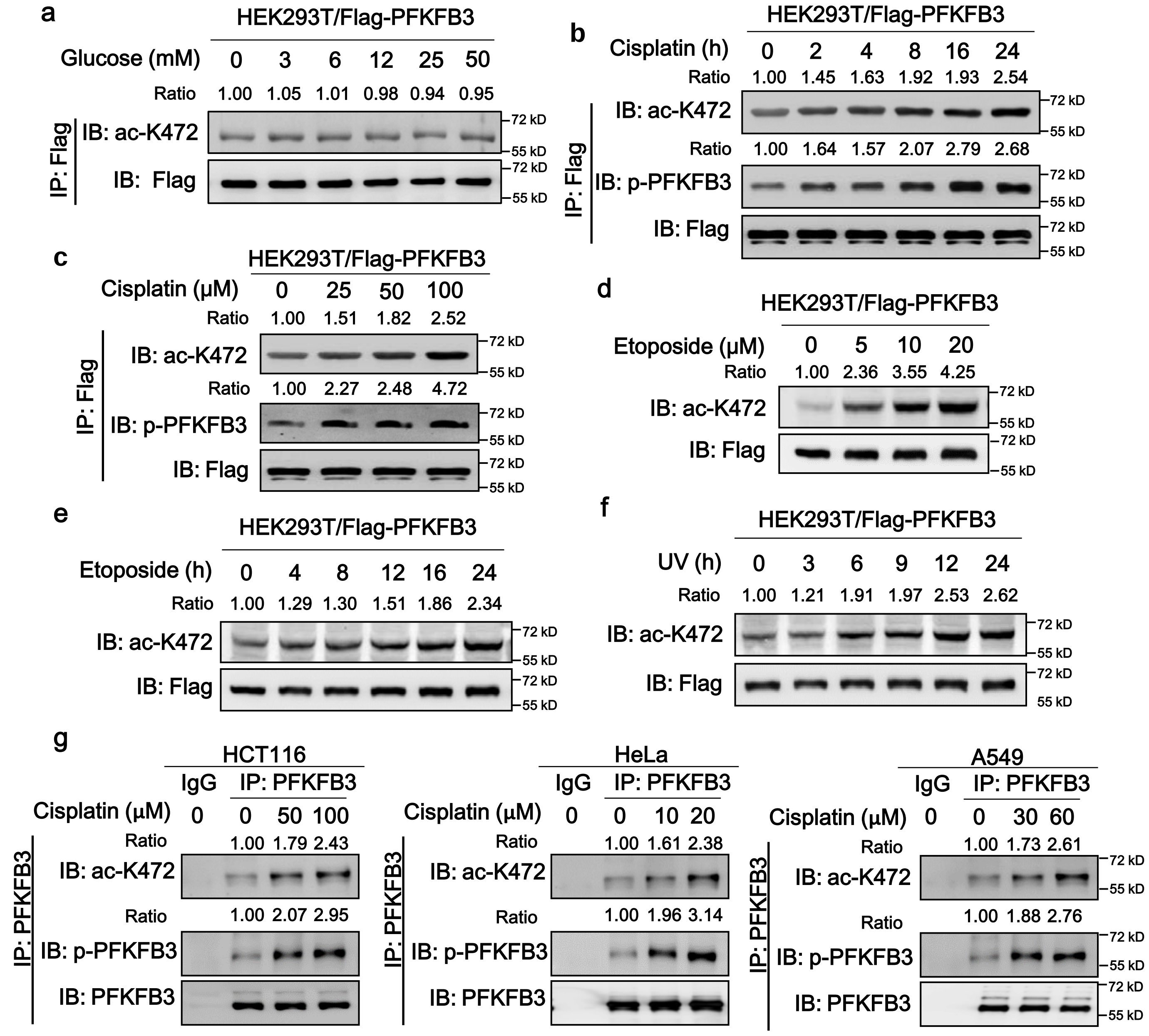

Supplementary Fig. 8 DNA damage signals induce the K472 acetylation of PFKFB3

(a)K472 acetylation of PFKFB3 was not sensitive to glucose concentration in the medium. Flag-tagged PFKFB3 was expressed in HEK293T cells, which were then treated with glucose at the indicated concentrations for 6 h. Flag-PFKFB3 was immunoprecipitated with Flag beads and immunoblotting was performed with the indicated antibodies. Relative PFKFB3 K472 acetylation was normalized by Flag protein.

(b, c) Cisplatin treatment increases K472 acetylation and S461 phosphorylation of PFKFB3 in a time- and dose-dependent manner. Flag-tagged PFKFB3 was ectopically expressed in HEK293T cells. Cells were treated with cisplatin for the duration indicated at a concentration of 50 μM (b) or at the concentrations indicated for 24 h (c).

(d, e) Etoposide treatment increases K472 acetylation of PFKFB3 in a dose- and time-dependent manner. Flag-tagged PFKFB3 was ectopically expressed in HEK293T cells. Cells were treated with etoposide for the duration indicated at a concentration of 10 μM (d) or at the concentrations indicated for 24 h (e). Immunoblotting was performed with the indicated antibodies.

(f) UV irradiation treatment increases K472 acetylation of PFKFB3 in a time-dependent manner. Flag-tagged PFKFB3 was ectopically expressed in HEK293T cells. Cells were treated with UV irradiation (10 J/m^2) and recovered for the duration indicated. Immunoblotting was performed with the indicated antibodies.

(g) Cisplatin induces K472 acetylation and S461 phosphorylation of endogenous PFKFB3. Endogenous PFKFB3 protein was purified from HCT116, HeLa and A549 cells after cisplatin treatment as indicated for 24 h. Immunoblotting was performed with the indicated antibodies. Relative PFKFB3 K472 acetylation and phosphorylation were normalized by PFKFB3 protein.

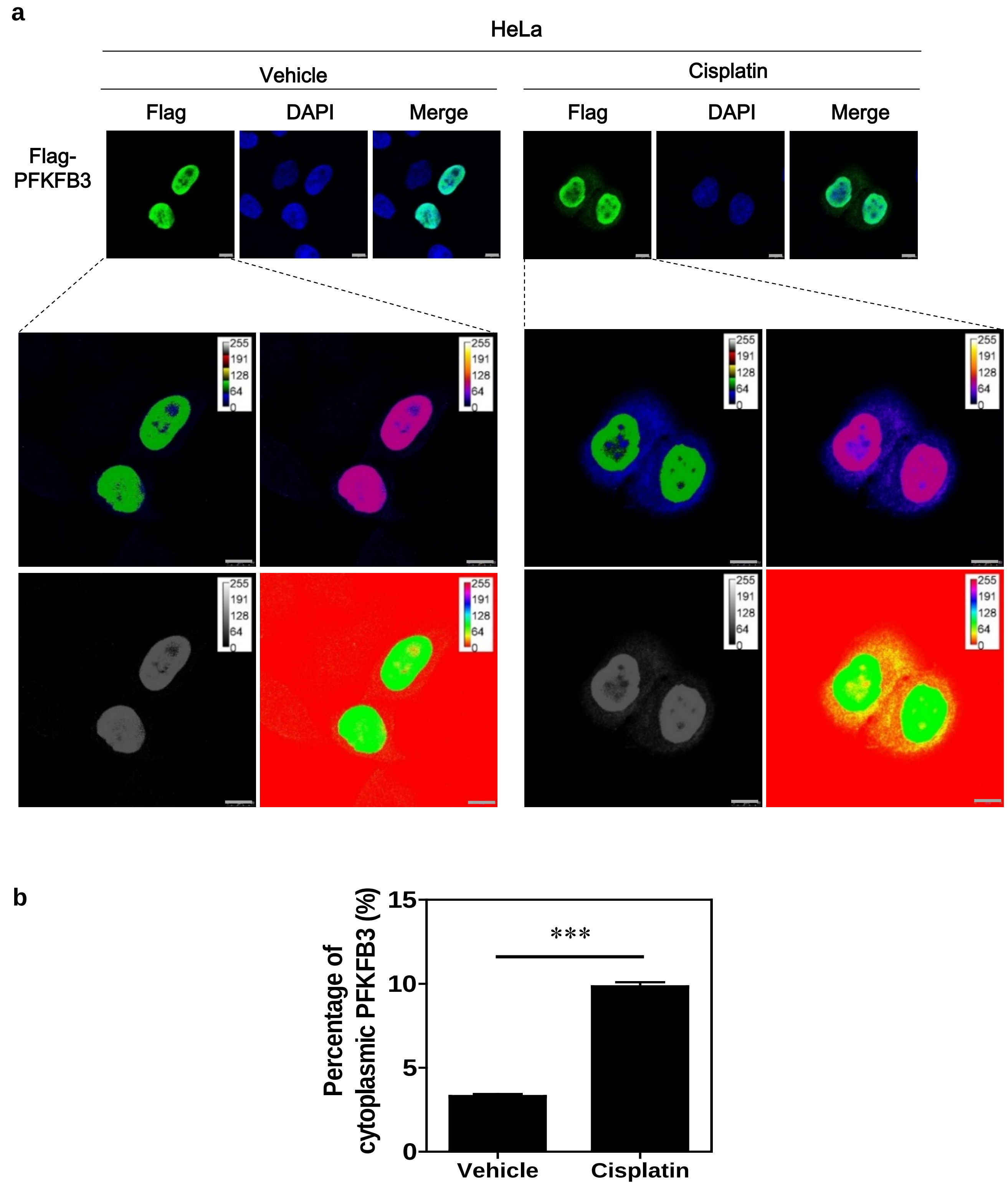

Supplementary Fig. 9 Cisplatin treatment induces PFKFB3 cytoplasmic accumulation

(a) Up panel: Flag-tagged of PFKFB3 was transfected into HeLa cells. After 24 h, cells were treated with or with cisplatin (10 μM) for 24h, followed by IF staining with anti-Flag primary antibody and then with Alexa Fluor 488-labeled secondary antibody (green). DAPI (blue) was used for nuclei staining. Low panel: The Flag channel of up panel is analyzed by Image J software and displayed with multi-color look up table (LUT). Scale bars: 10 μm.

(b) Quantification of cytoplasmic percentage of PFKFB3 described in (a). At least 100 cells were randomly selected and quantified for each group. Data are presented as mean ± s.d., and statistical analyses were performed using Two-tailed unpaired Student's t-test. *** denotes $p < 0.001$ for the indicated comparison.

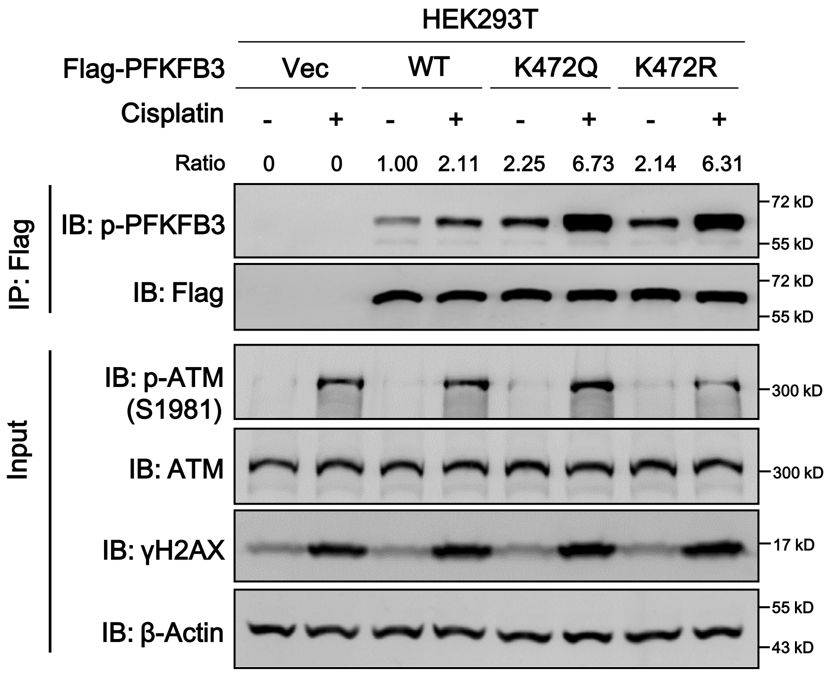

Supplementary Fig. 10 Cytoplasmic localization of PFKFB3 facilitated S461 phosphorylation induced by cisplatin. Flag-tagged WT, K472Q or K472R mutant of PFKFB3 was expressed in HEK293T cells. Cells were treated with cisplatin (25 μM) for 24 h before harvest. PFKFB3 was then immunoprecipitated with Flag beads, followed by immunoblotting with the phospho-AMPK substrate motif antibody. Phospho-ATM and γH2AX was examined to verify the effect of cisplatin treatment.

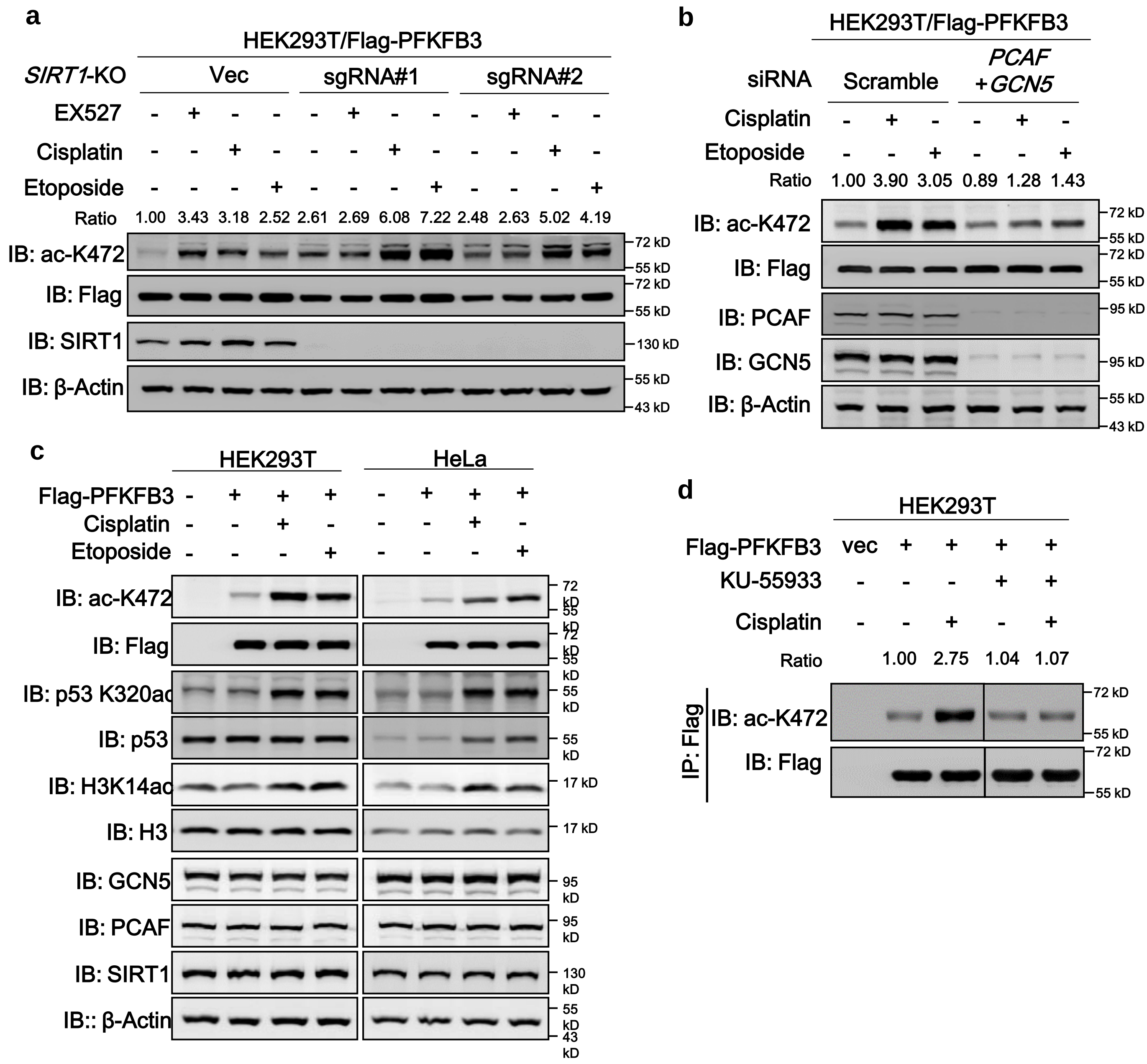

Supplementary Fig. 11 Cisplatin induced PFKFB3 K472 acetylation accumulation by increasing PCAF/GCN5 acetyltransferase activity.

(a) Cisplatin or etoposide treatment enhances K472 acetylation in *SIRT1* knockout cells. Flag-tagged PFKFB3 was expressed in WT or *SIRT1* knockout HEK293T cells, followed by treatments with EX527 (10 μM), cisplatin (50 μM) or etoposide (10 μM) for 24 h. Immunoblotting was performed with the indicated antibodies. Relative PFKFB3 K472 acetylation was normalized by Flag protein.

(b) Combined knockdown of *PCAF* and *GCN5* abolishes cisplatin or etoposide induced PFKFB3 K472 acetylation. Flag-tagged PFKFB3 was co-expressed with siRNAs targeting PCAF and GCN5 in HEK293T cells. Cells were then treated with cisplatin (50 μM) or etoposide (10 μM) for 24 h. Immunoblotting was performed with the indicated antibodies. Relative PFKFB3 K472 acetylation was normalized by Flag protein.

(c) Acetylation levels of GCN5/PCAF substrates were increased after cisplatin and etoposide treatment. Flag-tag PFKFB3 was ectopically expressed in HEK293T and HeLa , followed by treatment with cisplatin (50 μM for HEK293T and 10 μM for HeLa) or etoposide (10 μM for both cells) for 24 h before harvest. Immunoblotting was performed with the indicated antibodies.

(d) Inhibition of ATM abolishes cisplatin-induced PFKFB3 K472 acetylation. The HEK293T cells were transfected with Flag-PFKFB3 construct for 24 hr. These cells were pre-treated with or without ATM inhibitor (KU55933, 10μM) for 6 h, followed by treatment with or without cisplatin (50 μM) for 24 h. PFKFB3 protein was immunoprecipitated by Flag-Beads and immunoblotted with the indicated antibodies. Relative PFKFB3 K472 acetylation was normalized by Flag protein.

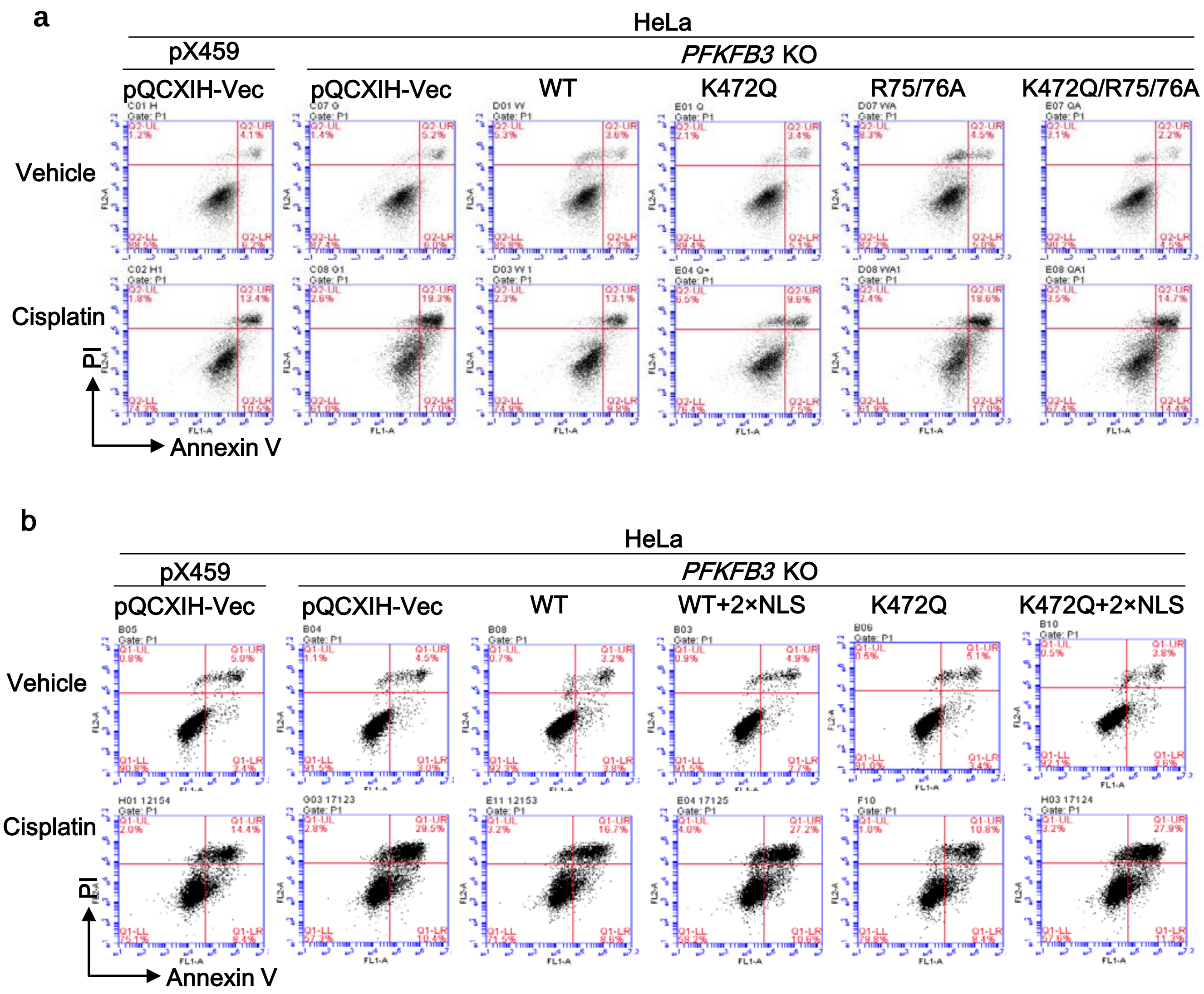

Supplementary Fig. 12 PFKFB3 protects cells from Cisplatin induced apoptosis

(a) Representative images of cell staining with by Annexin V and PI analyzed by flow cytometry as described in Fig. 7b.

(b) Representative images of cell staining with by Annexin V and PI analyzed by flow cytometry as described in Fig. 7h.

Supplementary Fig. 13 Uncropped images of western blot presented in above figures. Blue boxes show the region cropped from each blot and presented in above figures.

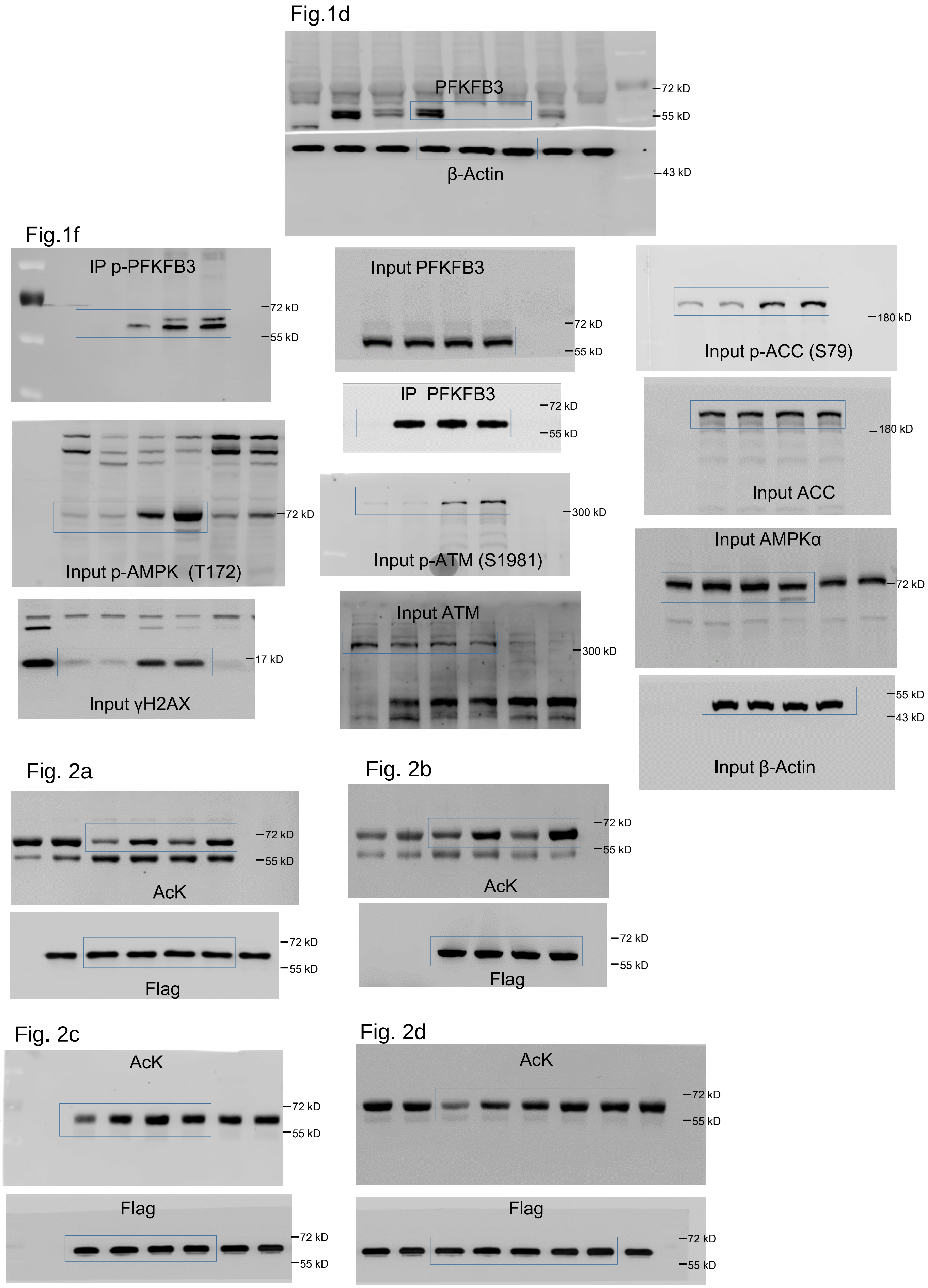

Fig. 2e

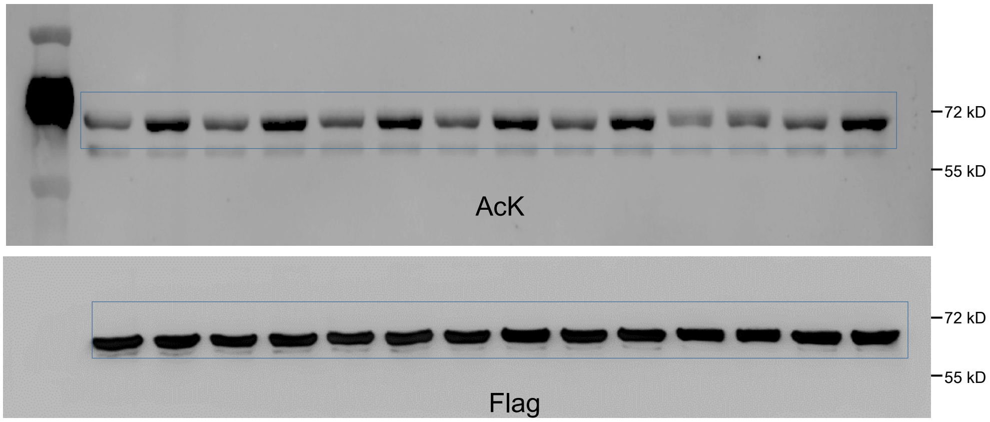

Fig. 2g

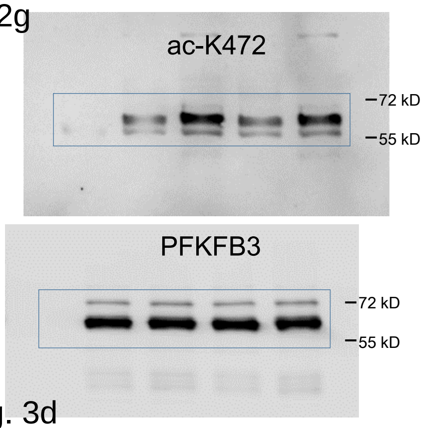

Fig. 2h

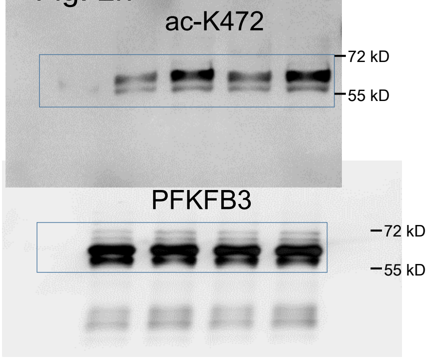

Fig. 3d

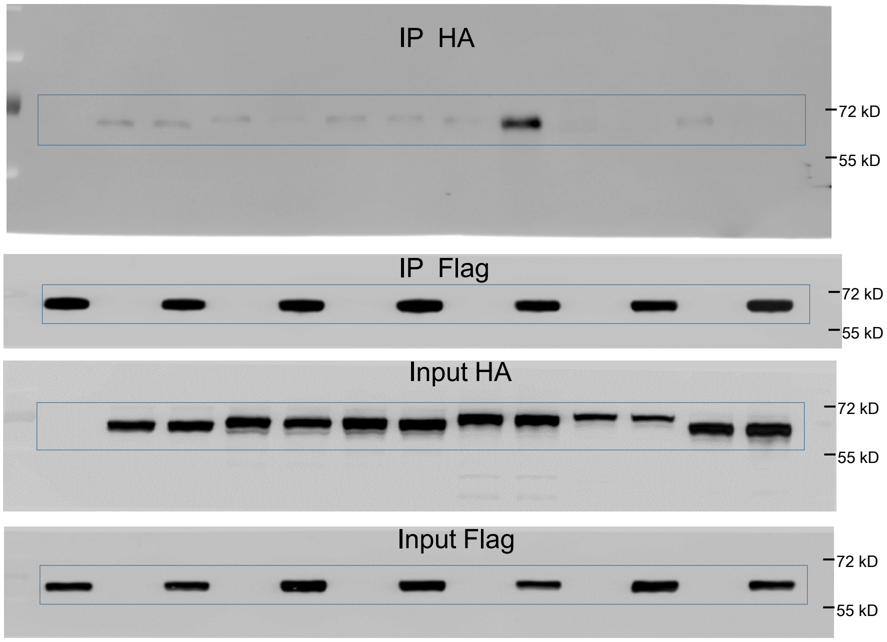

Fig. 3f

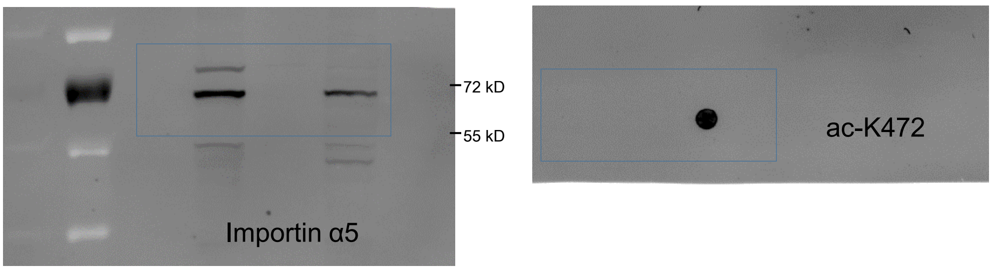

Fig. 3e

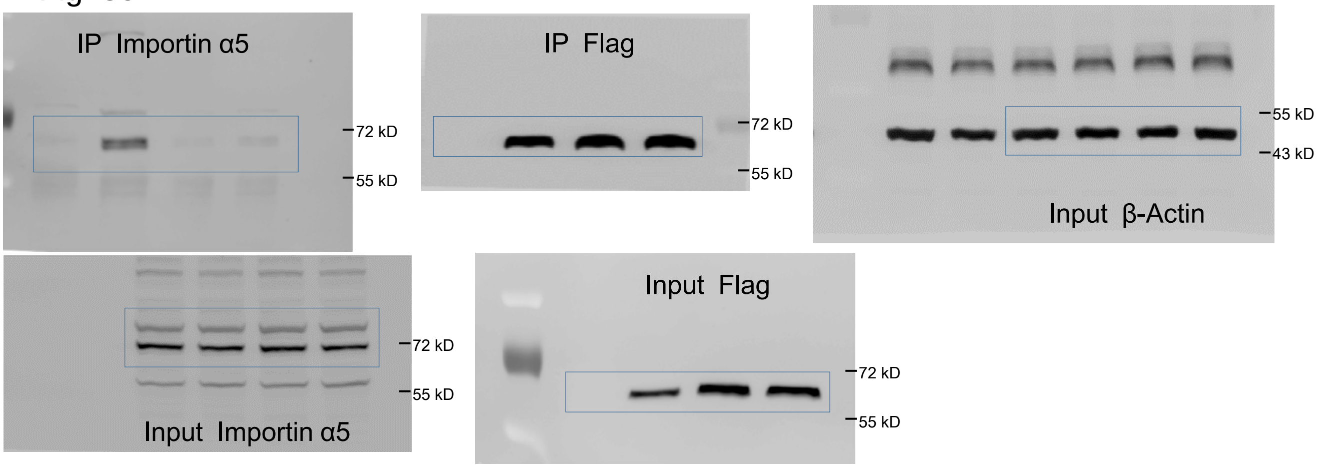

Fig. 4a

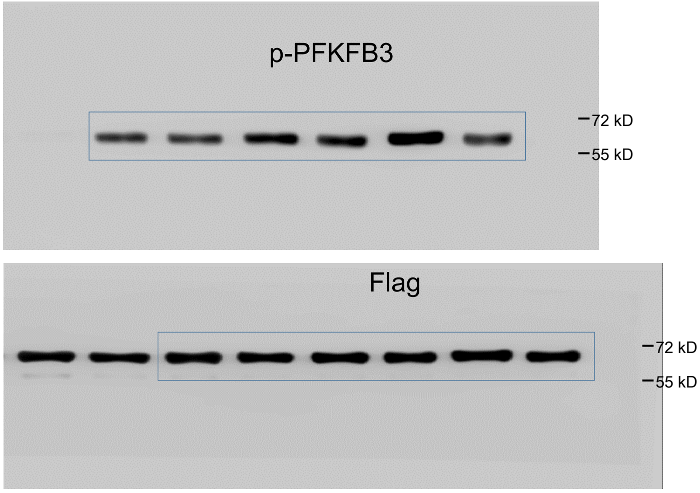

Fig. 4b

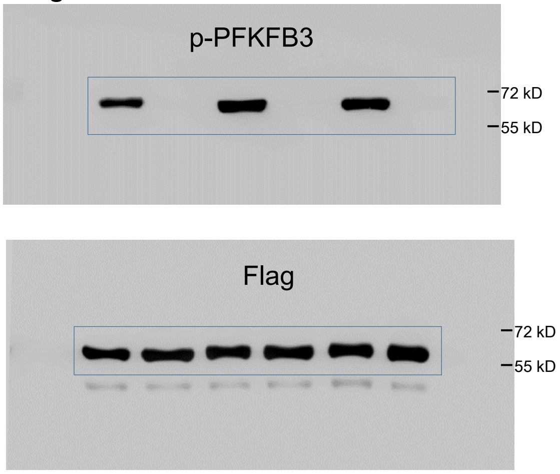

Fig. 4c

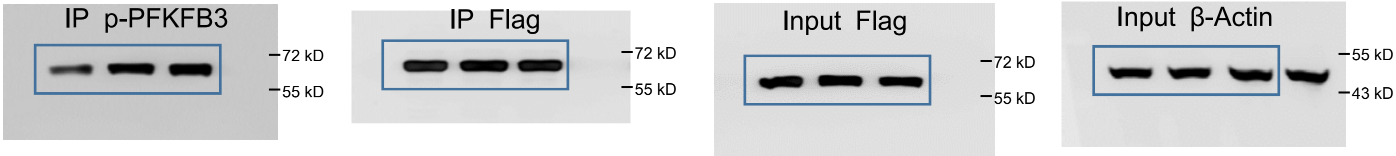

Fig. 4g

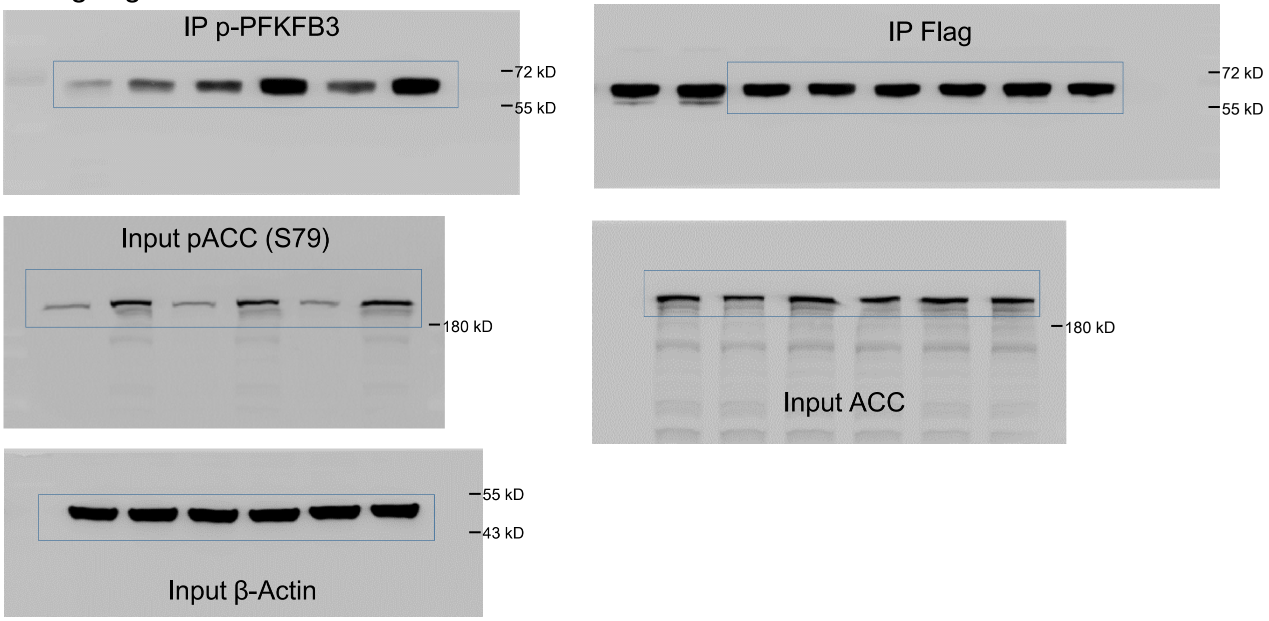

Fig. 5a

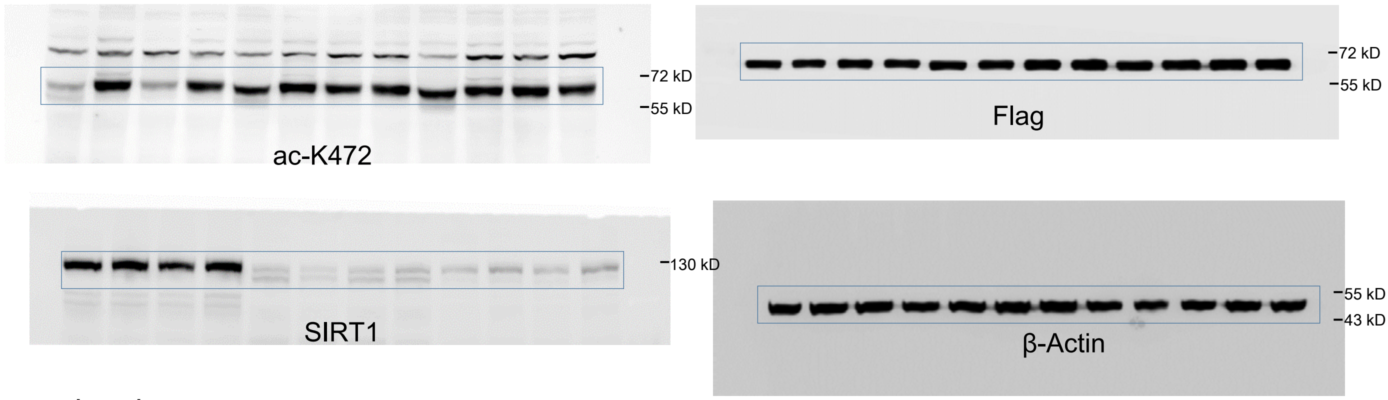

Fig. 5b

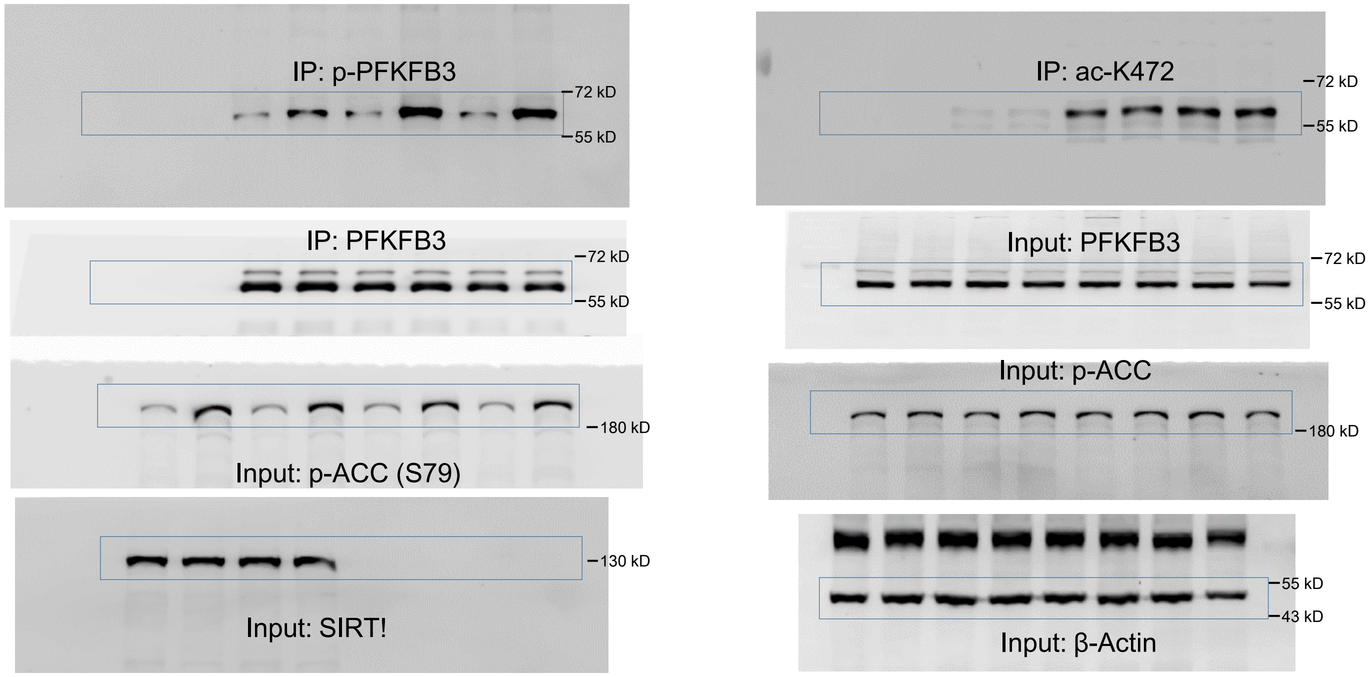

Fig. 5d

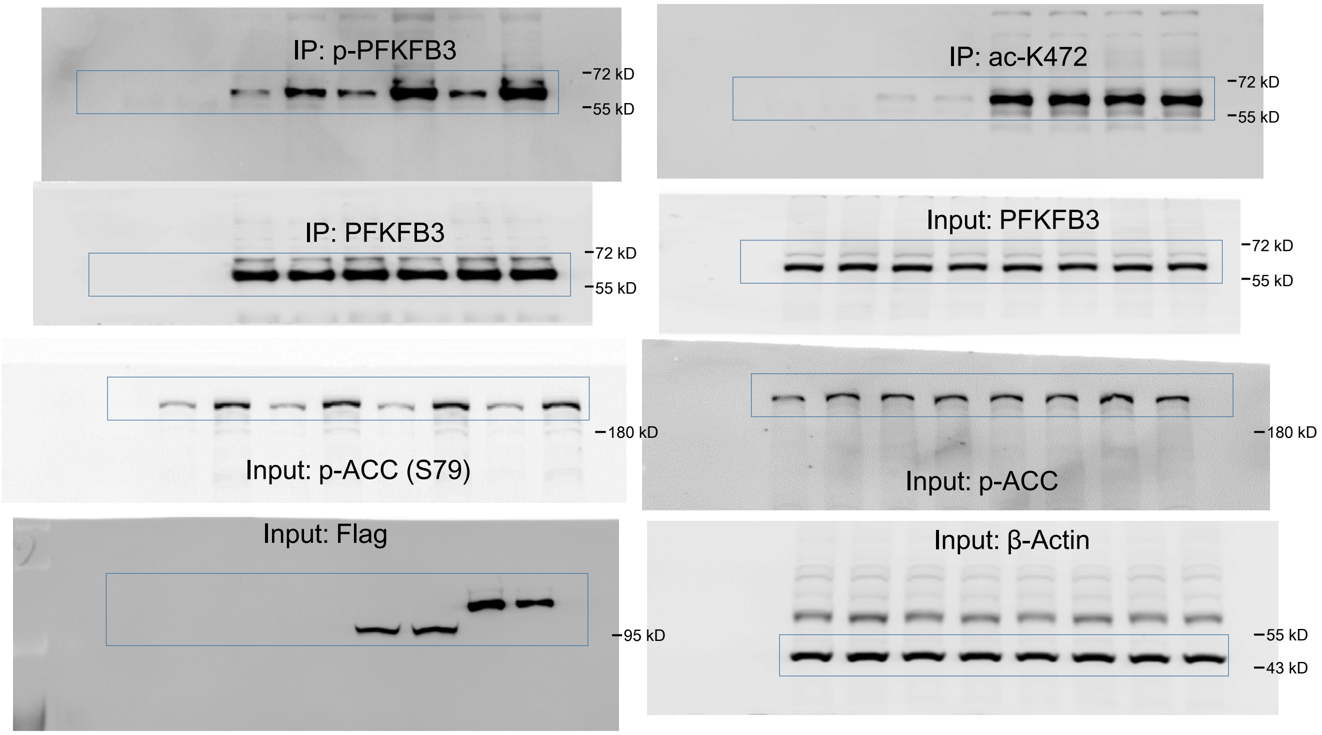

Fig. 5c

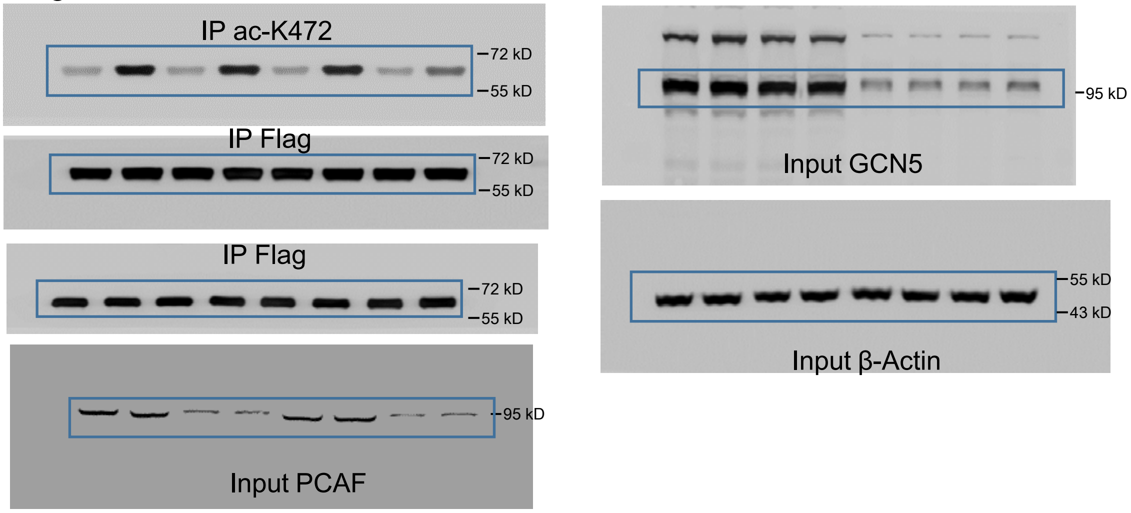

Fig. 6a

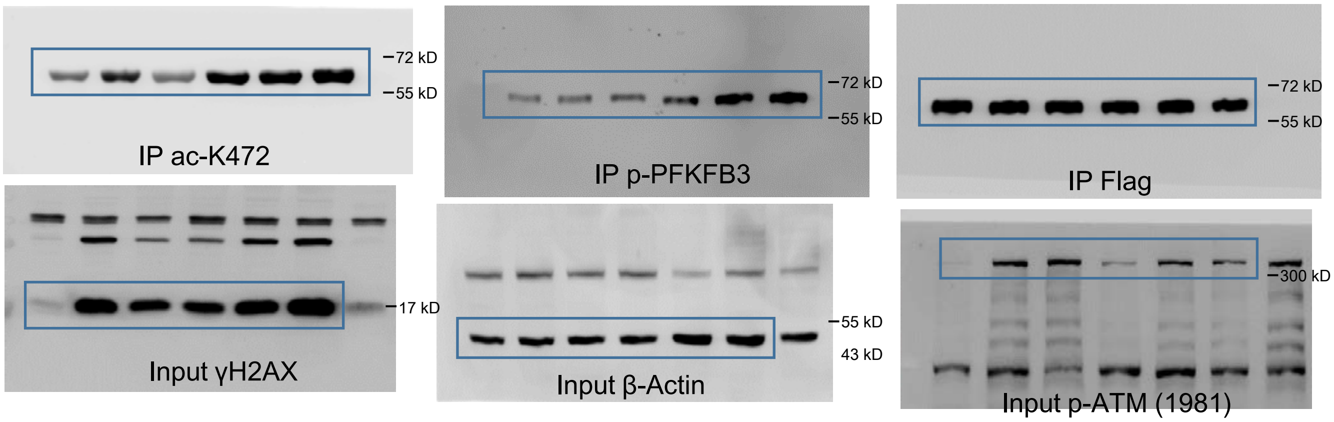

Fig. 6c

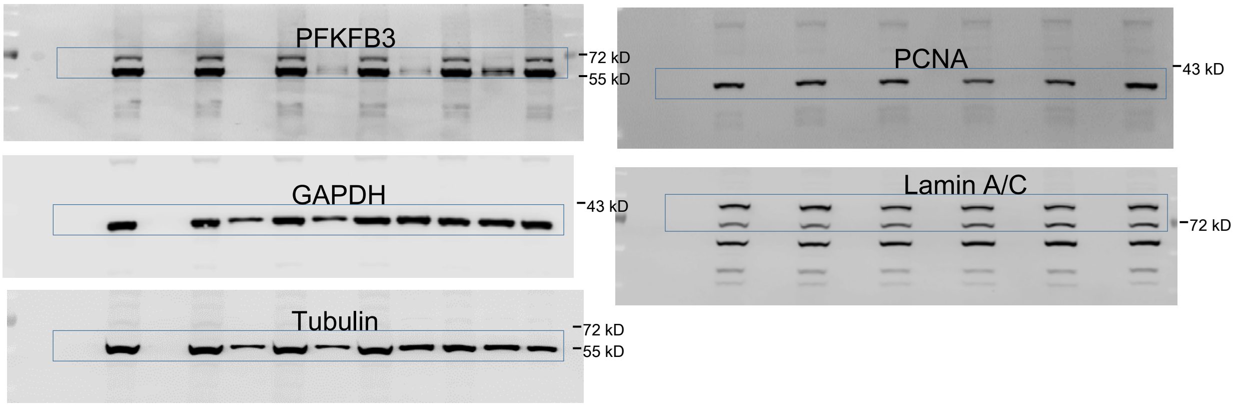

Fig. 6d

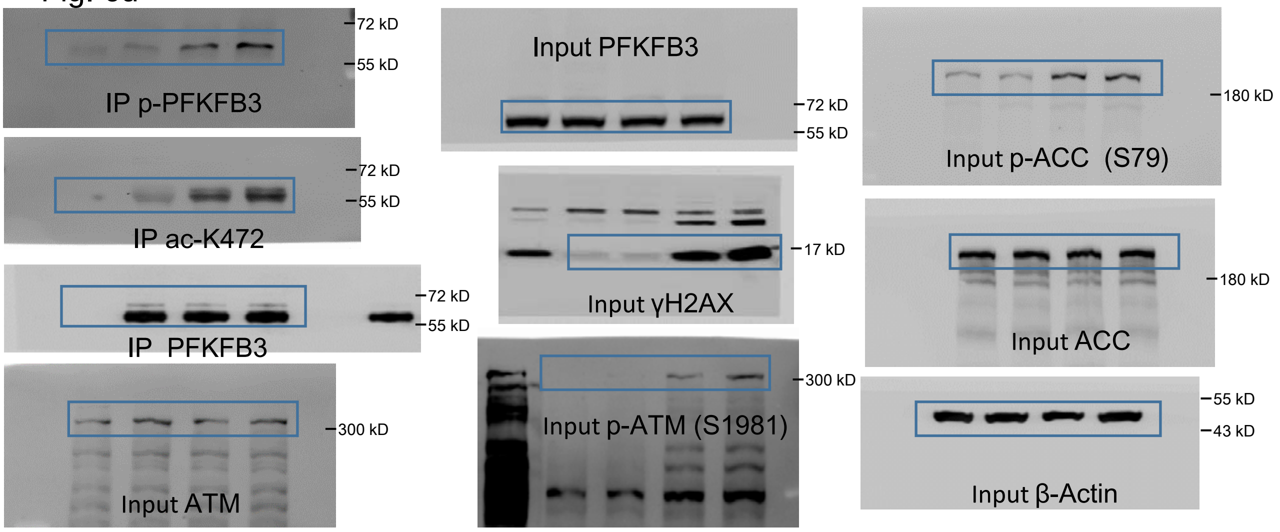

Fig. 6e

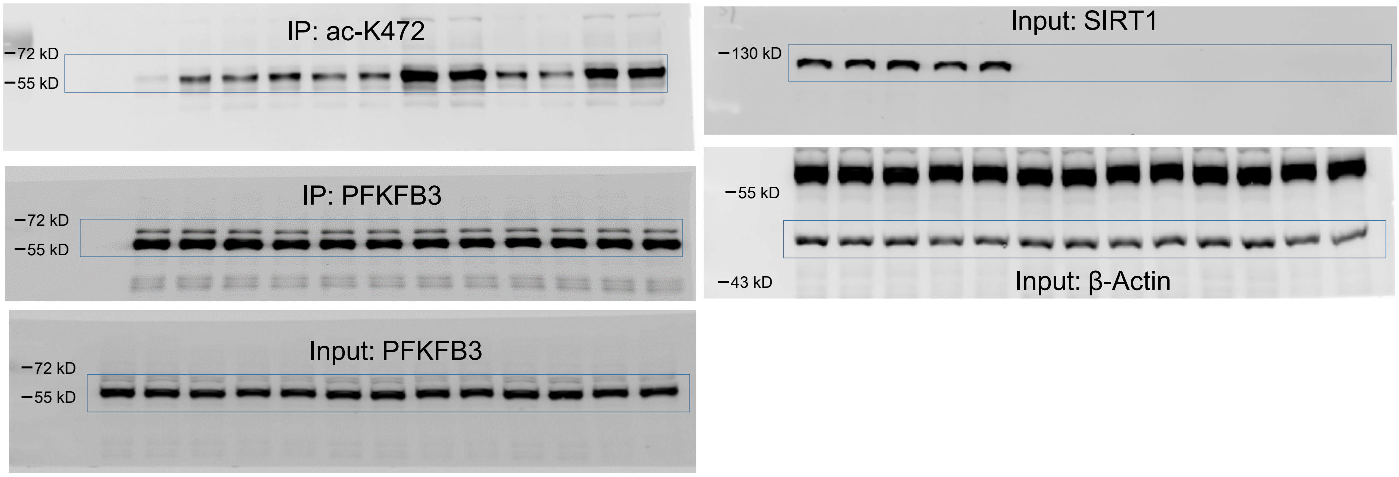

Fig. 6f

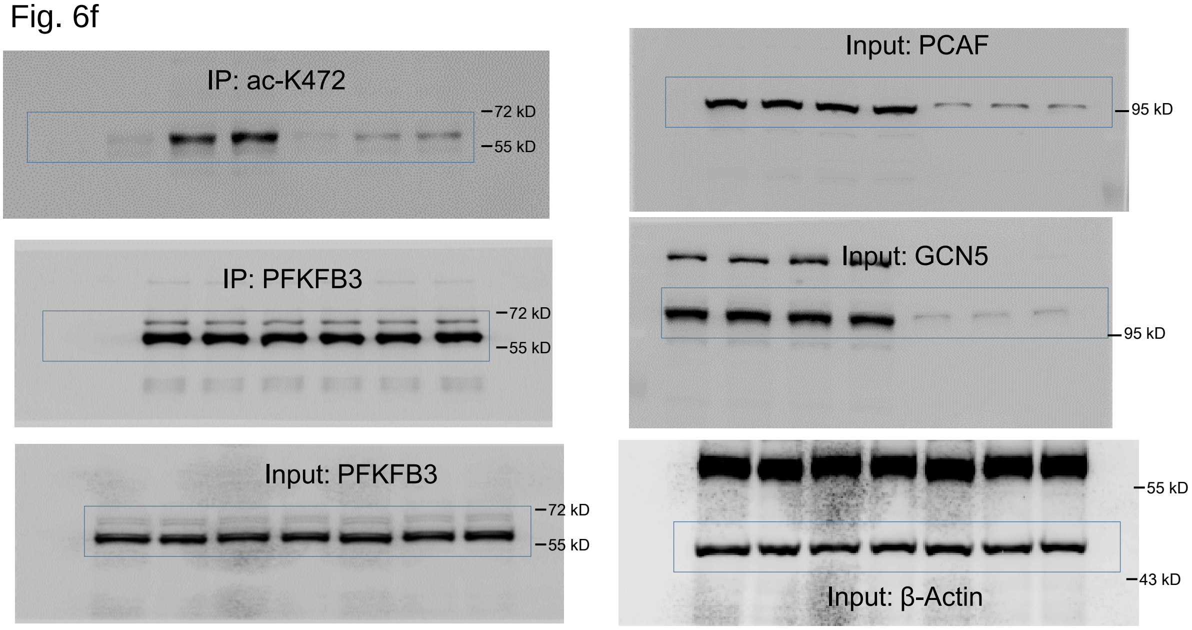

Fig. 6g

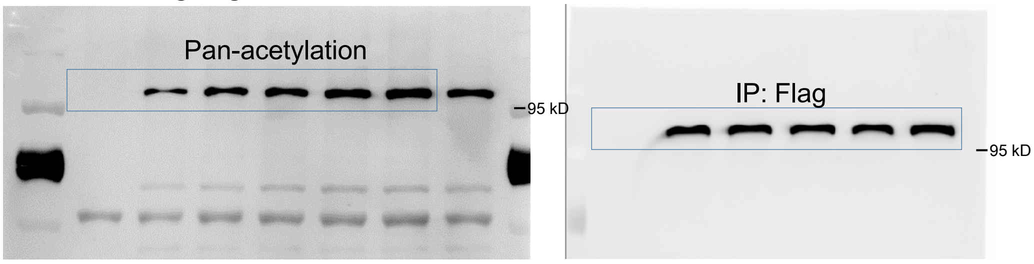

Fig. 6h

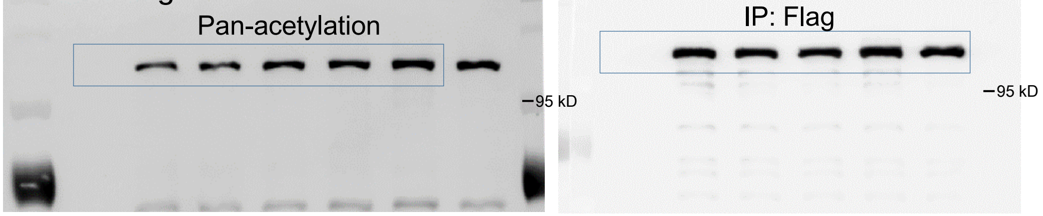

Fig. 6i

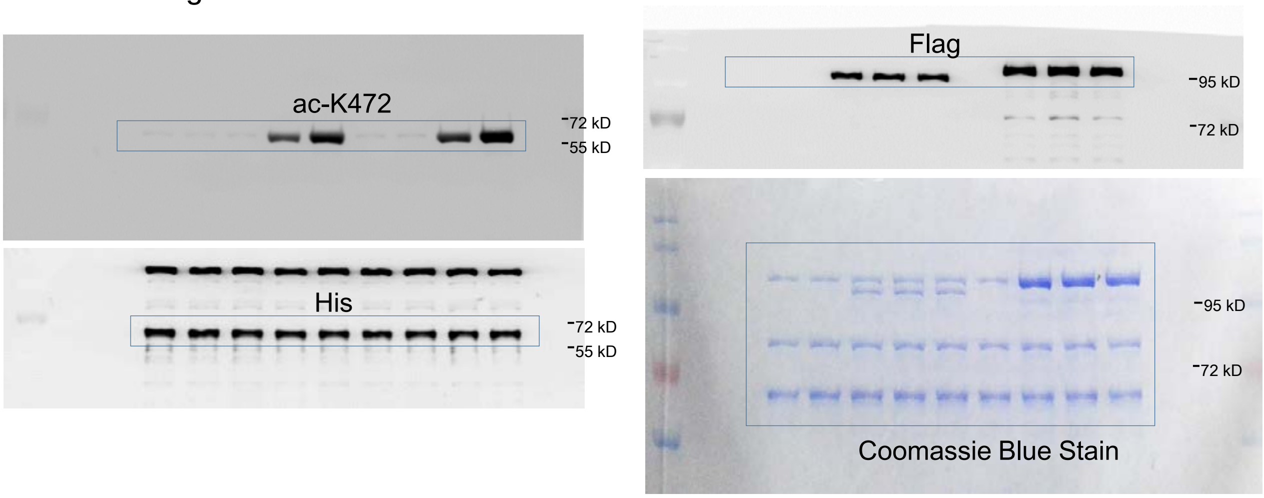

Fig. 7a

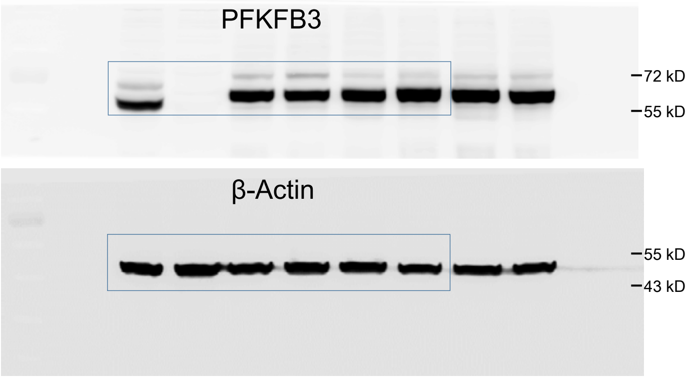

Fig. 7f

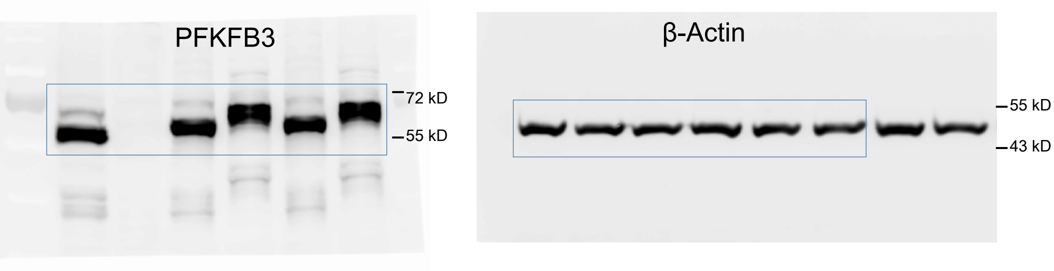

Supplementary Fig. 2c

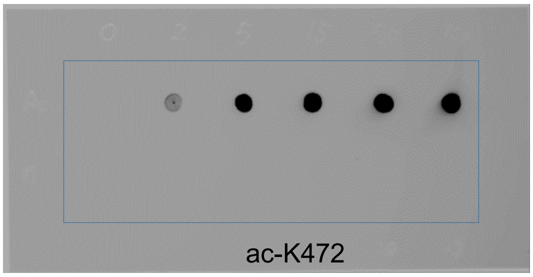

Supplementary Fig. 4

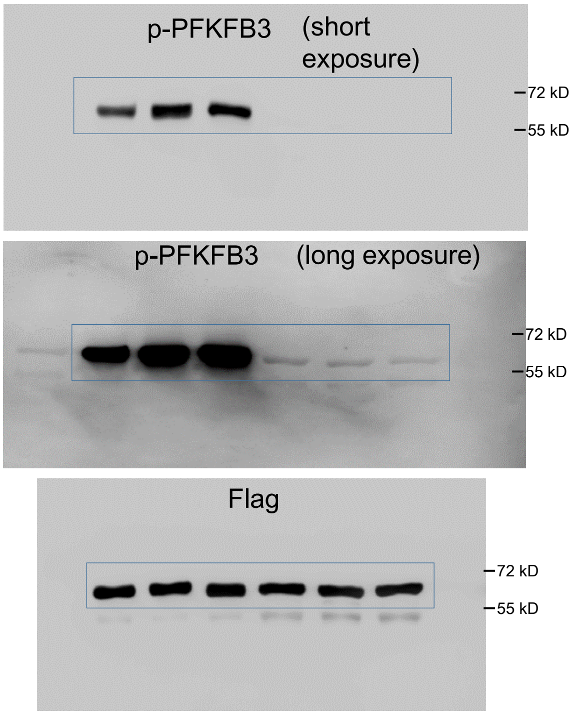

Supplementary Fig. 5a

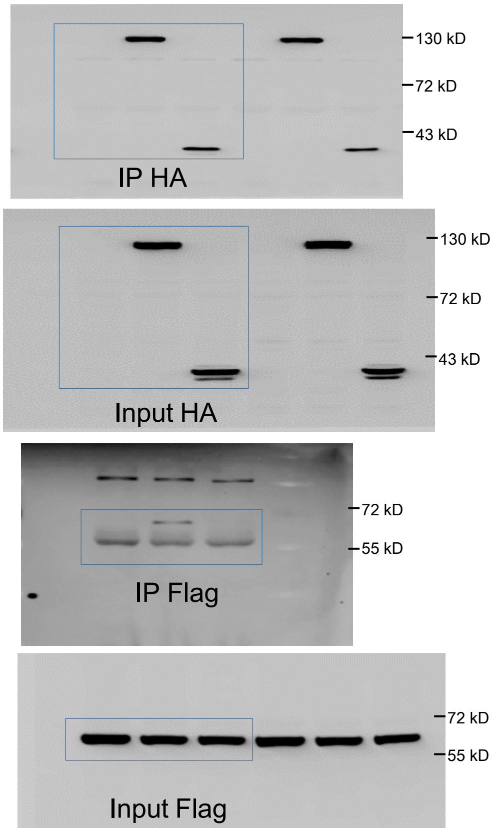

Supplementary Fig. 5b

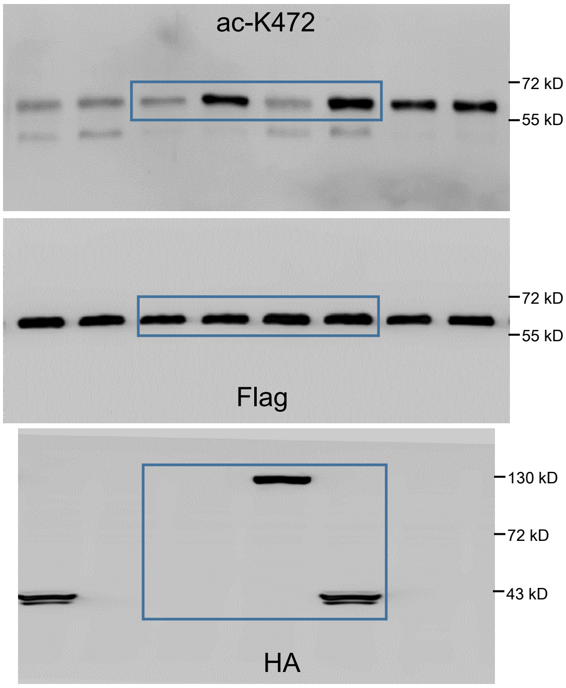

Supplementary Fig. 5d

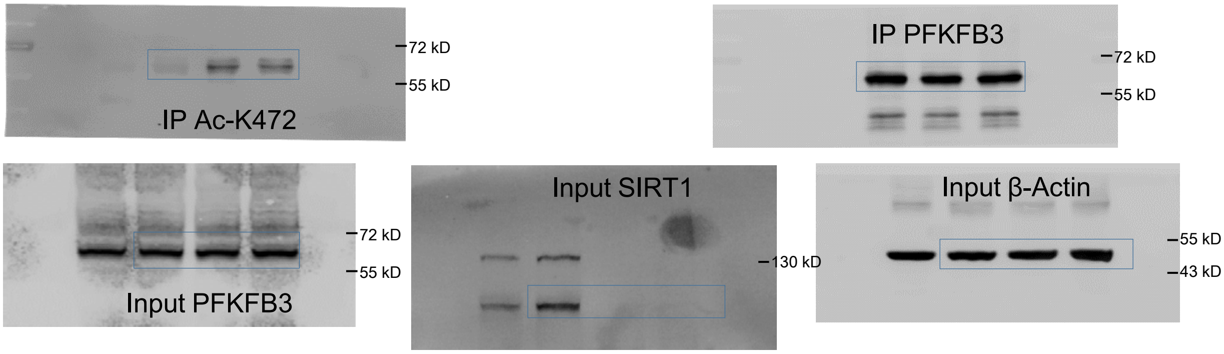

Supplementary Fig. 6a

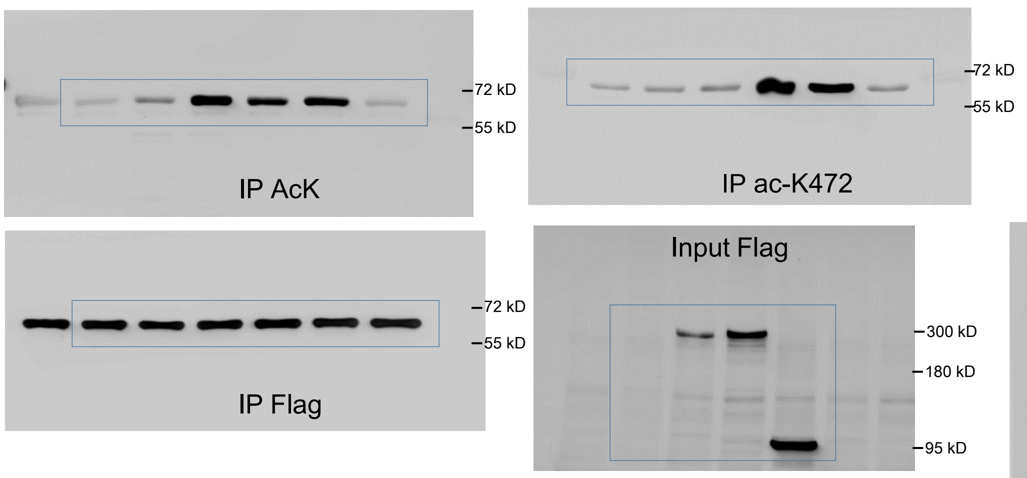

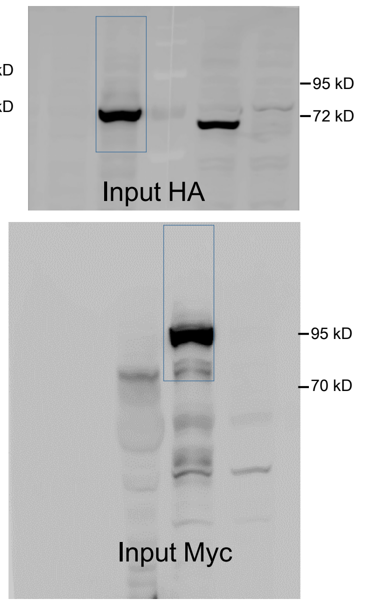

Supplementary Fig. 6b

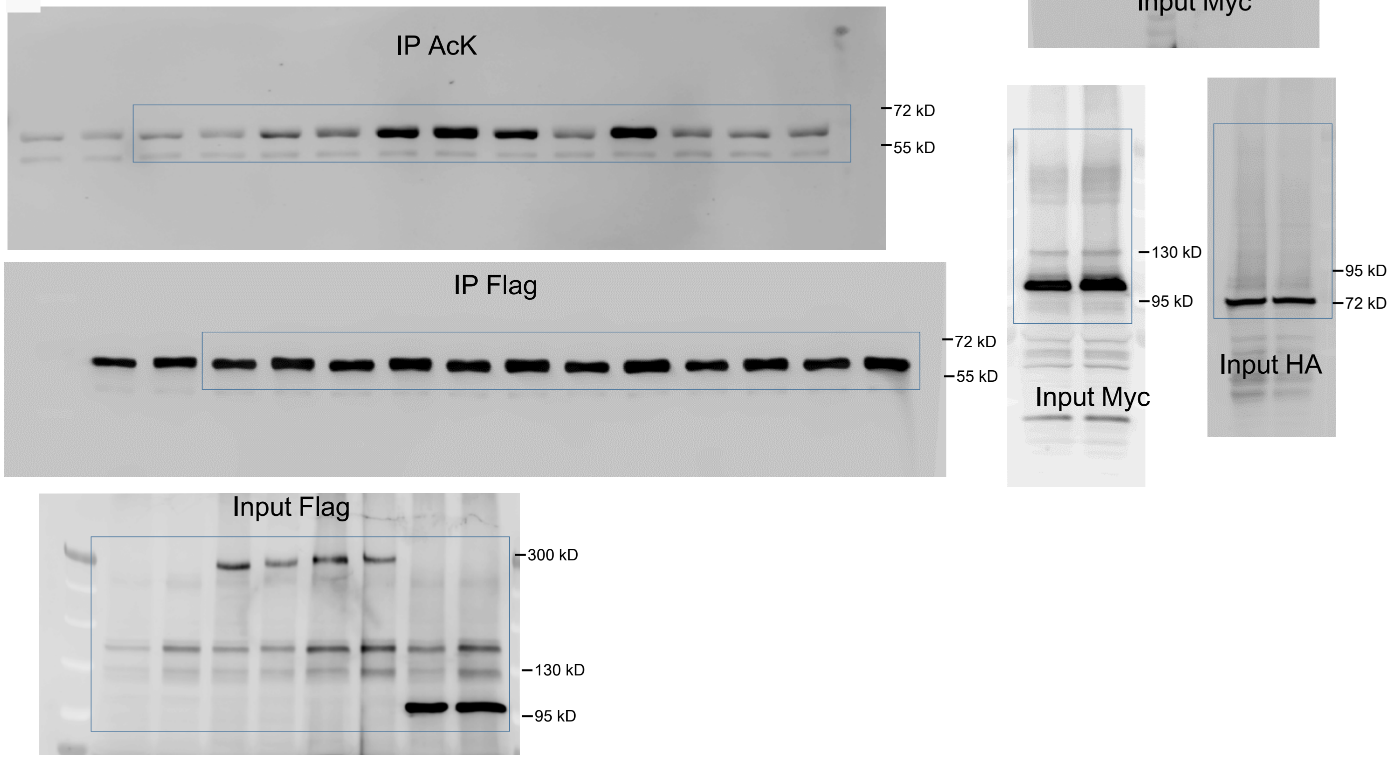

Supplementary Fig. 6d

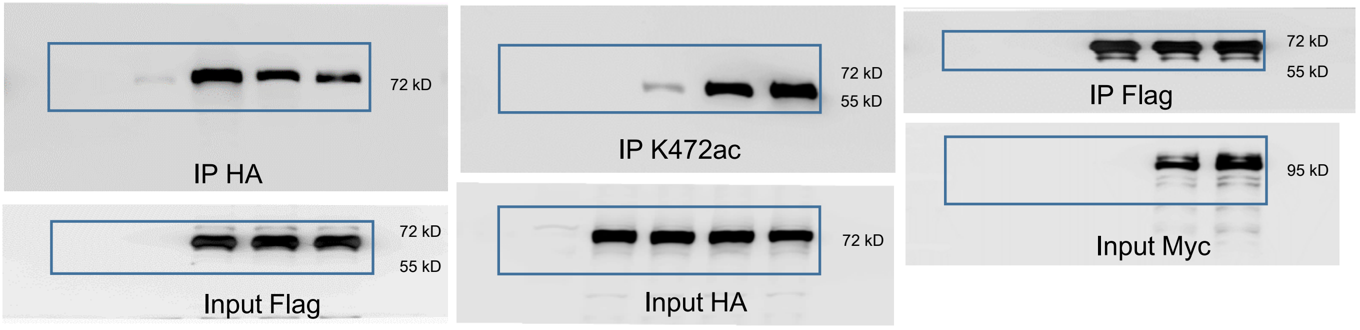

Supplementary Fig. 7a

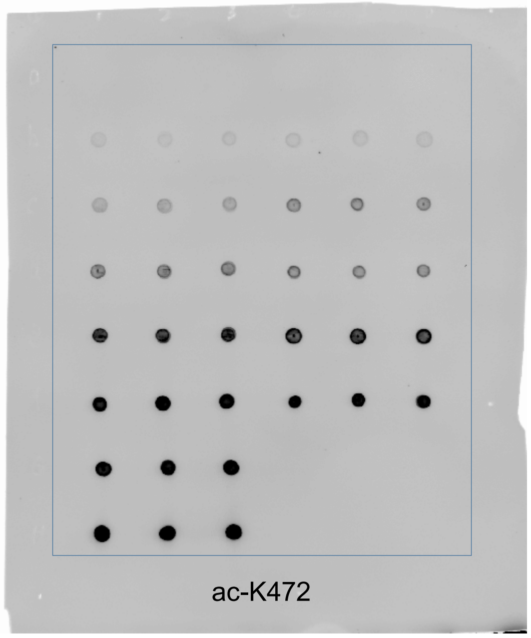

Supplementary Fig. 8a

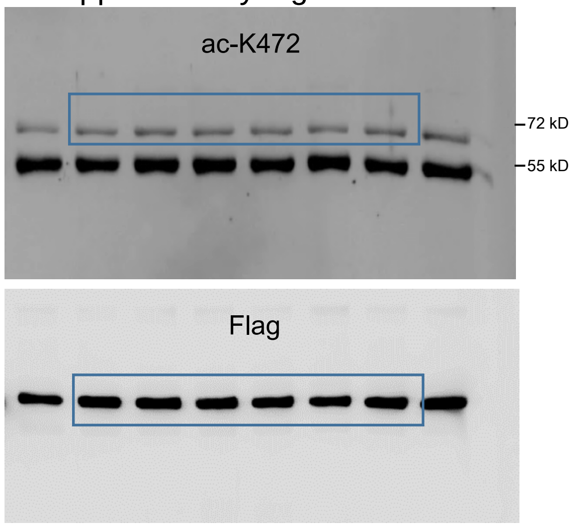

Supplementary Fig. 8b

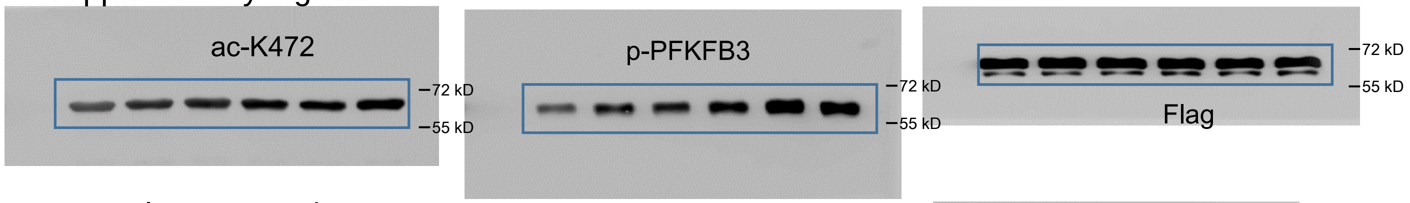

Supplementary Fig. 8c

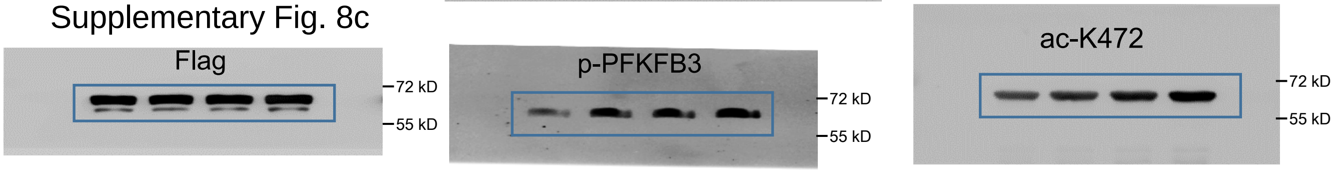

Supplementary Fig. 8d

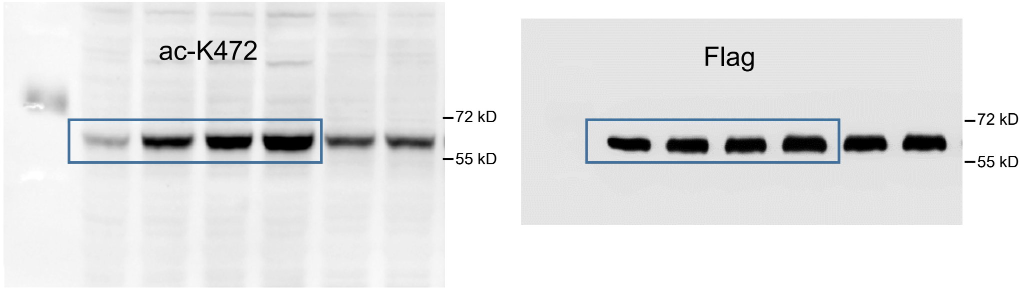

Supplementary Fig. 8e

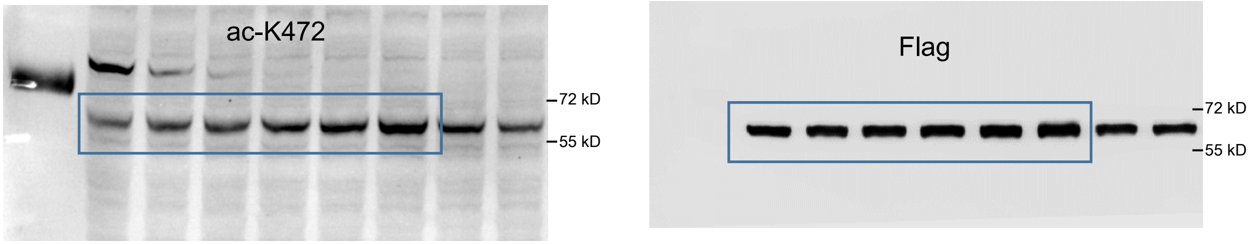

Supplementary Fig. 8f

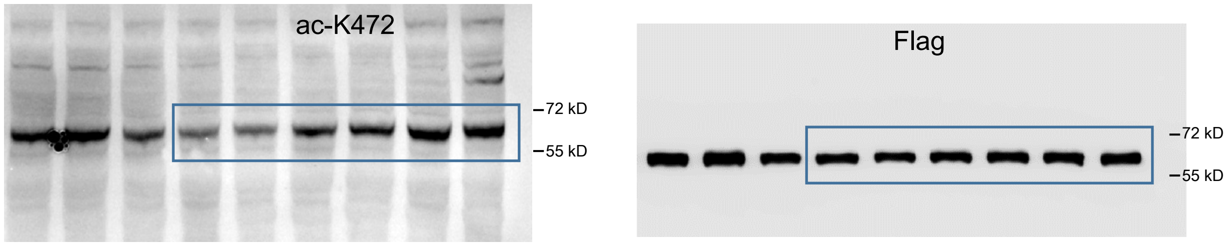

Supplementary Fig. 8g

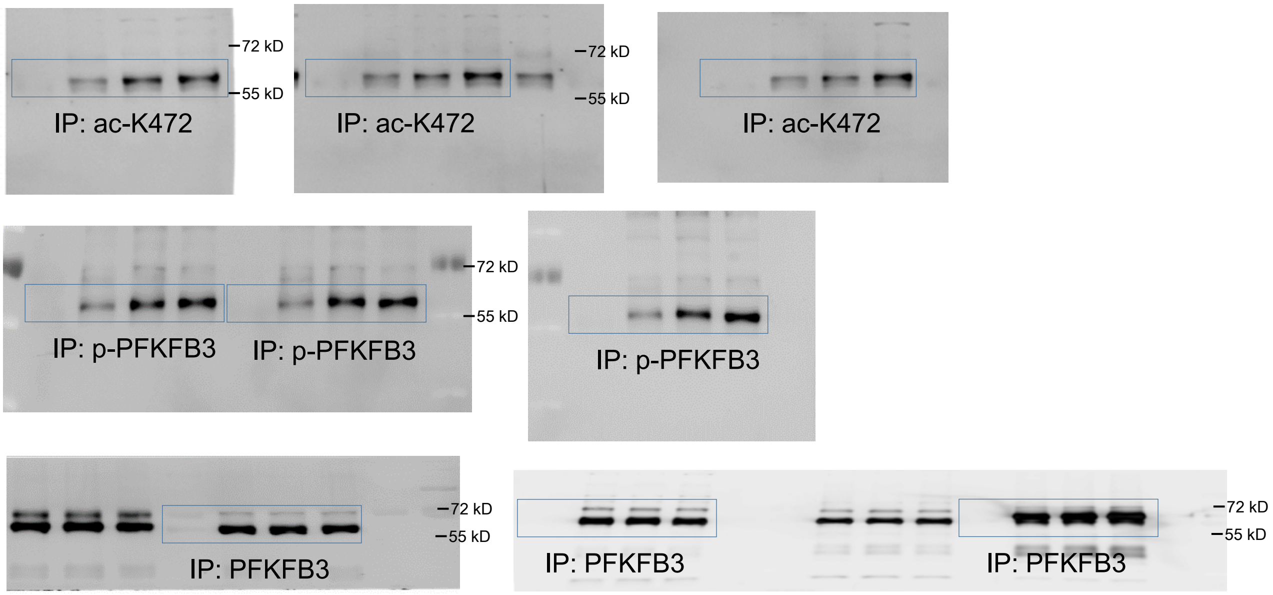

Supplementary Fig. 10

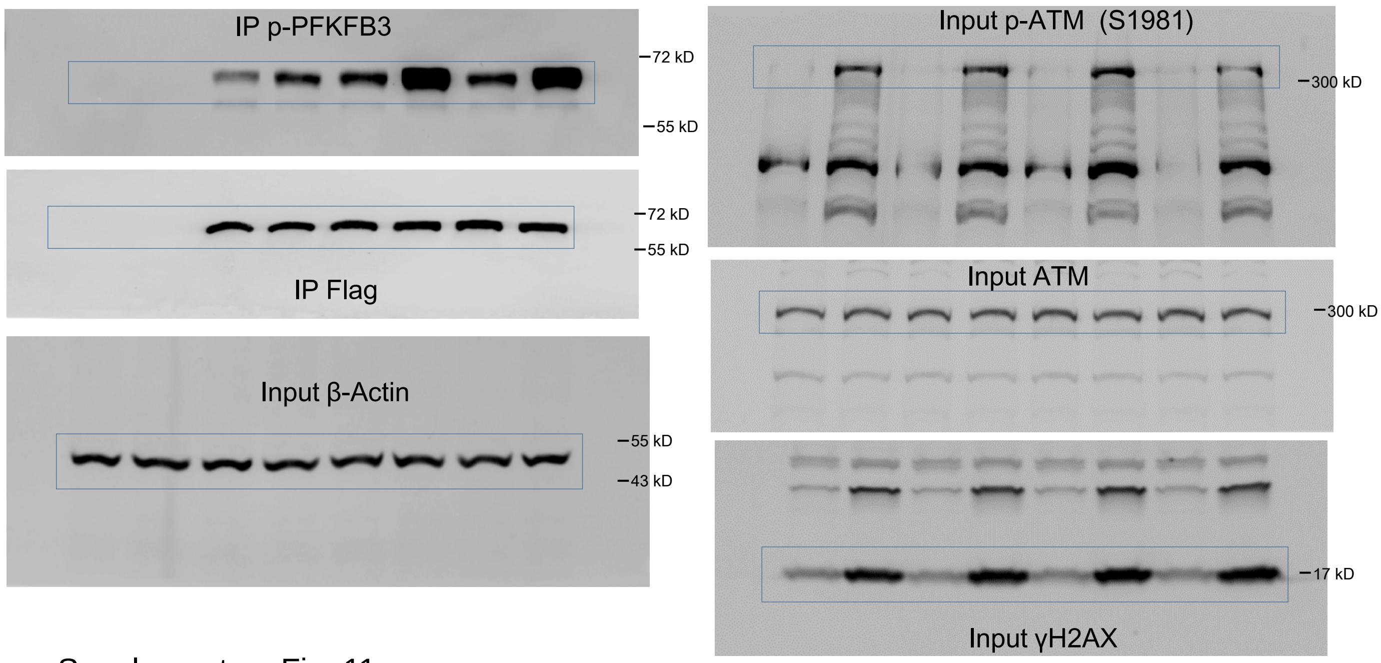

Supplementary Fig. 11a

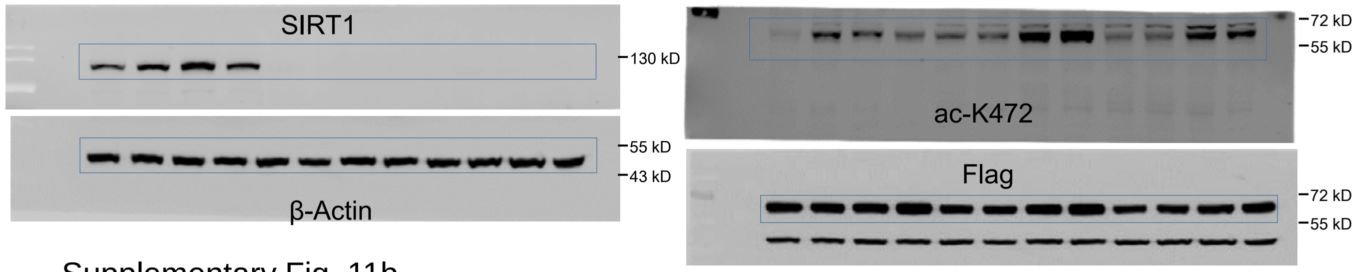

Supplementary Fig. 11b

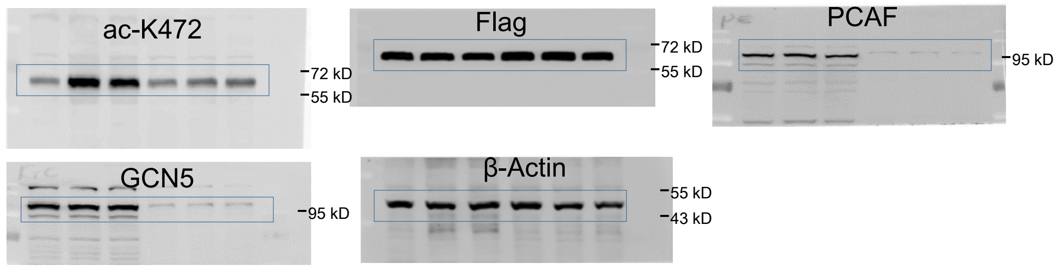

Supplementary Fig. 11c

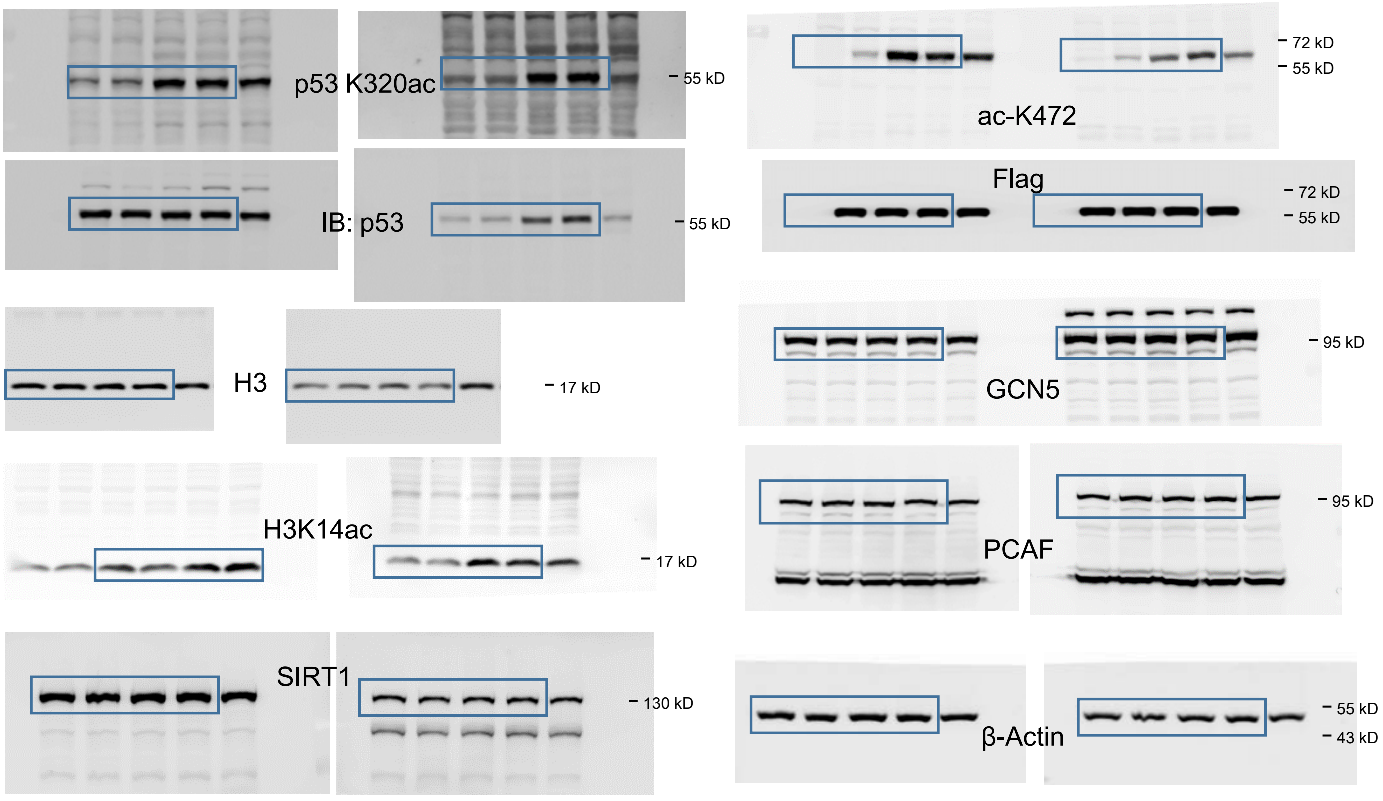

Supplementary Fig. 11d

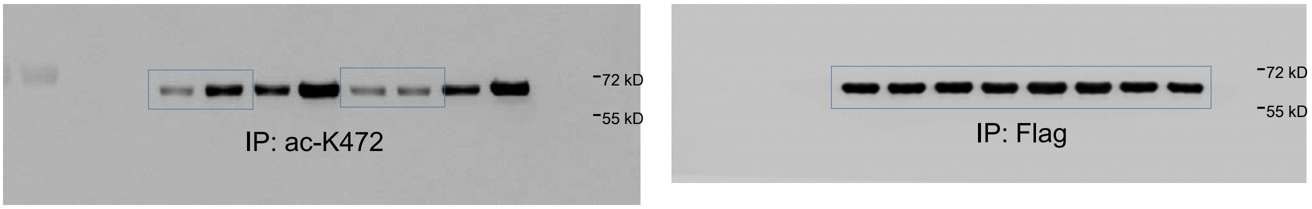

Supplement: Supplementary file 1 — Supplementary Information [file 41467_2018_2950_MOESM1_ESM.pdf]
